# Physical Activity Intervention for Loneliness (PAIL) in community-dwelling older adults: a randomised feasibility study

**DOI:** 10.1186/s40814-020-00587-0

**Published:** 2020-05-23

**Authors:** Anastasia V. Shvedko, Janice L. Thompson, Carolyn A. Greig, Anna C. Whittaker

**Affiliations:** 1 School of Sport, Exercise and Rehabilitation Sciences, University of Birmingham, Birmingham, UK; 2 MRC – Arthritis Research UK Centre for Musculoskeletal Ageing Research, University of Birmingham, Birmingham, UK; 3 Faculty of Health Sciences and Sport, University of Stirling, Stirling, UK; 4 NIHR Birmingham Biomedical Research Centre, University Hospitals Birmingham NHS Foundation Trust and University of Birmingham, Birmingham, UK

**Keywords:** Feasibility study, Physical activity, Loneliness, Older adults, Randomised controlled trial

## Abstract

**Background:**

Low quality social relationships in older adults are strongly associated with feelings of loneliness. Physical activity interventions could reduce loneliness and improve psychological well-being, among other health benefits. The aim of this study was to examine the feasibility of a Physical Activity Intervention for Loneliness (PAIL) in community-dwelling older adults at risk of loneliness.

**Methods:**

The PAIL feasibility study was a 12-week randomized controlled feasibility trial (RCT) conducted in Birmingham, United Kingdom, from February 2018 to August 2018, and ran in two waves of data collection. Eligible participants were community-dwelling adults aged 60 years and older, sedentary (less than 20 min of moderate-to-vigorous PA (MVPA) a week), and at risk of loneliness. The intervention included once-weekly group walk and health education workshop up to 90 min per session in total, with a wait-listed (WL) control group. The primary feasibility outcomes were to estimate recruitment, retention rates and adherence to the intervention. Secondary outcome measures (not blinded assessment) were body mass index, blood pressure, physical activity and psychosocial variables. Process and outcome evaluations were conducted using focus groups interviews. The recruitment and retention progression criteria for the definitive large-scale RCT was set a-priori.

**Results:**

Forty-eight participants were recruited over 4 months with a recruitment rate of 25% (48/195); 52% (25/48) met the inclusion criteria and 100% (25/25) were randomised into the intervention (*n* = 12) and WL control groups (*n* = 13). Participants were 25 older adults (mean (SD) 68.5(8.05) years), 14 (56%) female, and 18 (72%) white. At 12 weeks, 10/12 (83.3%) intervention and 10/13 (76.9%) control participants completed the final assessments. The average attendance rate was 58.3% for the intervention group (range 33.0%-75.0%) and 42.3% (range 23.1%–69.2%) among controls. The a priori recruitment and retention criteria for progression were not met. No serious adverse events occurred. The focus group results identified three themes which showed overall positive experiences of participation in PAIL in terms of (1) study design and intervention; (2) walking sessions; and (3) health education workshops.

**Conclusions:**

The findings suggest that community-dwelling older adults at risk of loneliness found the intervention and measures acceptable and could safely participate. However, a more extensive and robust strategy would be needed to support adequate recruitment of lonely older adults and adherence into a definitive RCT.

**Trial registration:**

Clinicaltrials.gov, NCT03458793

## Background

Maintenance of social connectedness throughout the lifespan is an important aspect of successful ageing [1]. The disruption of established social patterns or poor quality of social relationships is strongly associated with loneliness especially in older adults [2]. Defined as a discrepancy between a person’s desired and actual social relationships [3], loneliness and a lack of social relations are considered to be high risk factors for morbidity and mortality, and the negative impact of loneliness can be as harmful as smoking fifteen cigarettes a day [4]. Due to the deteriorating health condition of older adults and less ability to engage in social connections, the early prevention of loneliness in community programmes seems prudent.

Due to deteriorating health associated with ageing, older adults are highly predisposed to declines in cognitive and physical function [5]. However, regular exercise in older adults at the recommended minimum of 150 min of moderate PA per week performed in any length of bouts [6] can contribute to the maintenance of physical health. Additional benefits are associated with muscle strengthening and balance exercises performed in 10 min bouts for falls prevention [7]. Moreover, active older adults retain cognitive function at a high level throughout their older years, which is a very important aspect of social life and well-being [8, 9]. It is a health behaviour that can increase peripheral social networking and the acquisition of new social contacts due to engagement in a variety of physical and leisure activities by older adults outside the home. In turn, this can replace or compensate for lost social connections for older adults with feelings of loneliness, and turn these feelings into meaningful social connections based on the social compensation model [10]. PA improves psychological and emotional well-being leading to direct health benefits based on the so-called feel-good effect of exercise identified in the literature [11], which is associated with increases in serotonin, monoamine and neurotrophin production and reductions in the stress hormone cortisol [12].

Mechanisms of action of PA interventions are suggested to relate to loneliness reduction models, stress reduction and increased social support during activities. The social compensation model [13] suggests that PA can work via compensation for lost meaningful social connections due to increased peripheral social networking during friendly conversations between participants [14]. The broaden-and-build theory of positive emotions [15] posits that enjoyable forms of PA generate happiness and bring positive emotions, which in turn could be associated with loneliness reduction [16]. Based on the stress/social support model [17], social networks promote well-being that is associated with loneliness reduction in older adults. Finally, the tripartite model of group identification has been shown to be effective in group-based PA settings among specific population groups, such as lonely seniors, due to a sense of social identification [18, 19]. This model considers three aspects, such as cognitive (social categorisation, i.e. the degree by which an individual categorises him/herself as similar to other group members), affective (i.e. the degree to which an individual feels affectively attracted to the other group members), and behavioural (interdependence, i.e. the degree to which an individual evaluates his/her group as important for teaching objectives) [18, 19]. Through shared interests and goals during engagement in physical activities social activity is boosted and leads to group identification through the feeling of social attraction to other group members.

A previous review found that few PA interventions for loneliness reduction have been conducted in community settings [20]. This is also consistent with previous systematic reviews and meta-analyses [2, 18, 21, 22]. Results of meta-analysis performed for social functioning (as a sub-domain of health-related quality of life) in this review showed, that specific aspects of PA interventions can successfully influence social health [20] with the strongest effects being obtained for group setting exercise interventions, with delivery by a health/medical professional, and in a diseased rather than healthy population [20]. In addition, the majority of studies used a cross-sectional or longitudinal design, which does not allow determination of causality and limits the rigour of the research evidence [18]. Others assess loneliness as a secondary outcome within a number of other psychosocial outcomes, which limits the ability to fully examine the effectiveness of these interventions for reducing loneliness [23].

Further, a number of moderating, such as global [23, 24] and domain-specific self-efficacy [25] and mediating (driving the influence of PA on loneliness) factors, such as social support [26] between loneliness and PA may help to determine additional pathways of any PA intervention effects.

Bearing in mind the limitations of the current literature, understanding the mechanisms through which PA may reduce loneliness may bring new insights to the design of novel and effective PA interventions [18]. Further research is needed to explore the association between loneliness, self-efficacy and social support in the context of PA interventions for older adults. However, before the mechanisms can be fully understood, the practicalities and feasibility of implementation of such interventions with older adults should be tested in a feasibility trial [27] before proceeding to a definitive RCT. In order to assess participant experiences of such interventions, the present study utilised a mixed-methods research design, defined as the class of research where the researcher mixes or combines quantitative and qualitative research techniques, methods, approaches, concepts or language into a single study [28]. This research design can add valuable knowledge into a feasibility study. The aim of the study was to examine the feasibility of the Physical Activity Intervention for Loneliness (PAIL) intervention in community-dwelling older adults at risk for loneliness. For the planned future large-scale RCT, the primary hypothesis was that, compared with the inactive control group, participants in the intervention group would report a greater decrease in loneliness. The secondary hypothesis was that participants in the intervention group would significantly increase their amount of physical activity engagement per week, and this would be associated with greater positive changes in other psychosocial and health outcomes compared with the control group participants. The following specific aims of this feasibility study were to estimate:

1. Recruitment rate, attendance and retention rates (number of participants completing the study as a proportion of those randomised).
2. The acceptability of the intervention by participants, and willingness to participate.
3. The appropriateness of the statistical methods of data analysis used.
4. The required sample size for a future large-scale RCT derived from a power calculation.
5. The acceptability of measures, and the most suitable primary outcome measure for a future large-scale RCT.

In addition, to reflect the aims of a future large-scale RCT that this feasibility study was seeking to inform, the effect sizes between the intervention and control groups were examined; however, the analysis was exploratory due to feasibility studies not being adequately powered to test the effectiveness of the intervention [29].

## Methods/design

The full description of methods in available elsewhere [30]. A brief description is presented here. PAIL was a two-arm, 12-week randomised feasibility trial with a wait-listed control group delivered in Birmingham, United Kingdom, from February 2018 to August 2018, and ran in two waves of data collection. The trial was approved by the Science, Technology, Engineering and Mathematics (STEM) Research Ethics Committee of the University of Birmingham, UK (ERN_16-1419A). This feasibility study was guided by a populated CONSORT Extension to Pilot and Feasibility Trials (Additional file 1) and template for intervention description and replication (TIDieR) checklist [31] (Additional file 2). Written informed consent was obtained from all participants prior to entry into the study.

### Participants

#### Recruitment

Participants were recruited in two waves from September 2017 to April 2018, from the local neighbourhood (households) and communities in Birmingham via leaflets. Additional recruitment was facilitated during the eligibility screening [32]. Recruitment was aimed to be at a rate of 10 participants a month (to a minimum of 40 participants) for estimation of the critical parameters of the feasibility study [33].

Initial eligibility was phone-based with the further eligibility screening conducted at the university centre. After providing informed consent, participants were invited to a presentation meeting about the study. This was delivered at the university by the main researcher (AS), and included a detailed description of the project aims, methods and procedures, and a Question and Answer session. Attendees were invited for further eligibility screening at the university. Potentially eligible participants identified after baseline screening were randomised into the intervention or a WL control group using a computer-generated random sequence performed by an external researcher not involved in the delivery of the intervention or outcome assessment. Participants were informed about the group allocation by e-mail or a phone call by a person not involved in assessments or delivery of the intervention. At the outcome assessment level, participants who were assessors of their own psychosocial outcomes using questionnaires, were blinded to their group allocation at the time of completing the initial questionnaires. Intervention providers who were responsible for outcome assessments were not blinded to the intervention delivery as this would not be possible, given that the PhD student researcher (AS) conducted the study and walks.

#### Eligibility

Participants were eligible if they were (1) community-dwelling older adults aged 60 years and older; (2) previously sedentary (i.e. less than 20 min of moderate-to-vigorous PA (MVPA) per week over the past month) [34]; (3) at risk of loneliness and having ≥ 6 out of 9 points on the 3-item loneliness scale during the phone screening [35]; (4) physically mobile as measured using the short physical performance battery (SPPB) [36] with a score ≥ 9 out of 12 [37]; (5) having chronic diseases but ambulatory; (6) able to give written informed consent; and (7) English speaking and able to complete paper and pencil questionnaires. Exclusion criteria were as follows: < 60 years old, regularly physically active or already engaged in another PA intervention, socially active, having a disease that seriously precluded participation in PA, having a cognitive impairment as assessed by the Montreal Cognitive Assessment (MOCA) [38] with a score ≥ 22 out of 30 [39] and not English literate.

### Intervention development and delivery

The development of the PAIL intervention was a result of collaborative work of the research group based on the characteristics of effective interventions obtained from our previously published systematic review [20]. The theory of active engagement [40] influenced the choice of moderators such as social support and self-esteem through an acquired sense of purpose and confidence during enjoyable forms of PA. The walking group leader attended a training course focused on exercise for older adults “Move it or lose it” [41] and was a certified group exercise instructor. The intervention was a group walking intervention with an education workshop on healthy ageing topics alongside each walking session once per week. After the pilot of the entire intervention with five people and feedback obtained at focus group interview conducted in March 2018, minor changes were needed to modify the delivery approach of the intervention. Firstly, it was suggested to facilitate the recruitment of participants by contacting the Birmingham 1000 elders group [42] and the BVSC consortium [43] to advertise the intervention for the summer period. Due to a small number of participants per group, the intervention was lacking the necessary social interactions between participants. Therefore, it was suggested to add new participants who were eligible to join the current groups, and identify the start date of their 12-week intervention from the day they joined (i.e. on a rolling basis). Participants received a weekly e-mail with information about the walking route and a topic of the workshops to set appropriate expectations and help them prepare for the discussion. Weekly information about social events was added to support local engagement with activities and facilitate within group social support. Additionally, free access was gained to Winterbourne House and Gardens (https://www.winterbourne.org.uk/) to deliver a healthy workshop, which included free beverages.

### Interventions

The PAIL feasibility study was a 12-week intervention consisting of group walks and health educational/social interaction workshops performed once weekly for a duration of up to 90 min per session. The design and features of the PAIL intervention were derived from the findings from a systematic review of PA interventions for loneliness by Shvedko et al. [20]. The theory of active engagement [40] influenced the choice of moderators such as social support and self-esteem through an acquired sense of purpose and confidence during enjoyable forms of PA. The PAIL was a face-to-face intervention delivered in small groups (up to 8-9 people per group) by a trained walk leader certified in group exercise for older adults and run in various locations and parks in Birmingham, UK. Prior to the first walking session, participants received a copy of a General Practitioner (GP) letter to inform their doctor of participation. Walking sessions were based on the principles of gradual progression and adaptation to PA [44]. The intensity of the walks was light-to-moderate and was monitored objectively by heart rate monitors using the age-predicted heart rate maximum (HR_max_) method [45] and subjectively using the talk test [46] and the 0-10 Borg Ratings of Perceived Exertion scale (RPE) [47]. Participants had to talk back comfortably during exercises using the talk test [46], and rate their RPE from 2 to 4 [47]. Participants followed a trained walking leader via a specific route (Additional file 3). A warm-up preceded each session followed by an end of session cool-down and breathing exercises. Group walking sessions were followed by health education/social interactions workshops on a variety of healthy ageing topics such as eye hygiene, mental health and well-being, preventing falls, social support, nutritional guidelines, and PA recommendations for older adults where participants shared their knowledge and experiences about the topics discussed.

#### Intervention group

After randomisation, participants in the intervention group started the 12-week intervention.

#### Wait-listed control group

Participants in the WL control (delayed intervention) group started the intervention after their follow-up measures were completed, approximately 12 weeks post-randomisation.

### Measures

All measures were conducted at the host academic institution at baseline and immediately post the intervention period. Participants provided socio-demographic information about their age, gender, ethnicity, marital status, living arrangements, level of education, any children, employment status and any medical conditions. *Cognitive function* was assessed using a Montreal Cognitive Assessment scale (MOCA) designed to test mild cognitive impairment [38]. *Physical functioning* was assessed using the short physical performance battery (SPPB) [36]. *Height* was measured to the nearest 0.1 cm using a stadiometer (Seca AG, Reinach, Switzerland) and recorded in metres. *Weight* was assessed using weighing scales (Tanita UK Ltd., Middlesex, UK) to the nearest 0.1 kg. *Resting blood pressure* (BP rest, mm Hg) was measured using a portable semi-automatic OMRON sphygmomanometer (OMRON HEM705CP sphygmomanometer; Omron Matsusaka Co Ltd, Japan). *Physical activity* was measured using ActivPAL accelerometers (PAL Technologies Ltd. Glasgow, UK) at baseline and immediately post intervention over a continuous 7-day period of awake and sleeping (24 h a day) except when bathing or swimming [48].

#### Questionnaires

*Loneliness* was assessed using the 8-item UCLA Loneliness Scale (UCLA-8) [49]. *Social support* was assessed using the 20-item Medical Outcomes Study Social Support Survey (MOSSSS) [50]. *Social networks* were categorised using the 6-item Lubben’s Social Network Scale (LSNS-6) [51]. *Depression and anxiety* were assessed using the 14-item Hospital Anxiety and Depression Scale (HADS) [52]. *Self-efficacy for exercise* was measured using the revised 9-item Self-Efficacy for Walking/Exercise Scale (SEE), using a paper-and-pencil format [53]. *Satisfaction with level of social contacts* (SSC) was measured with the question “How satisfied are you with your social contacts?” [54]. *Expected outcomes and barriers for exercise* were measured using the Expected Outcomes and Barriers for Habitual Exercise scale [55] adapted for the older adult population. Four questions related to sport competence were deleted from the expected outcomes sub-scale due to irrelevance for this population group [55]. The expected outcomes and barriers for exercise scale has demonstrated good internal consistency from 0.66 to 0.85, and a high test-retest reliability of 0.78 in previous research [55].

#### Qualitative assessments

To understand participants’ experiences of taking part in the PAIL feasibility trial, focus groups were conducted at the mid-point (between week 4 and 5) and at the end of the 12-week intervention using semi-structured discussions in groups of 4-9 people per group of mixed gender (Additional files 4, 5) on the following topics: reasons for participation, progress on intervention delivery and possible barriers to attending. The research team iteratively analysed the mid-point data to identify if any alterations in the intervention were required based on the participants’ feedback. Focus groups were audio recorded using a digital recorder and transcribed verbatim. An independent trained focus group leader acted as moderator and facilitator of the focus groups [56].

#### Feasibility outcomes

The following specific aims of this feasibility study were assessed:

1. Attendance was calculated as the total number of attended sessions divided by the total number of sessions of the intervention and recorded as a percentage.
2. Recruitment rate was calculated as the number of individuals responding to advertisements and friends’ referrals out of a total number of formal invitations given/advertisements placed (including web-based advertisements, advertisements placed in the local cohort groups and poster and leaflet material disseminated in the community). Recruitment rate was recorded as a percentage, e.g. 25% (48/195). It is acknowledged that advertisements may have reached a larger number of individuals, but it was impossible to quantify this.
3. Retention rate was calculated as number of participants completing the study as a proportion of those randomised.
4. The assessment rate of questionnaires was evaluated as the total number of completed questionnaires divided by the total number of questionnaires and recorded as a percentage.
5. The suitability of the statistical methods of data analysis was investigated using reliability analyses. Internal consistency reliability (Cronbach’s alpha) was calculated at each time point (pre and post) and averaged to give overall reliability. The expected outcomes and barriers for exercise questionnaire were completed twice at baseline, with a week between measures, to allow for test-retest reliability analysis.
6. The acceptability of the intervention by participants, and willingness to participate was assessed using focus group. The focus group transcripts were analysed using a phenomenological inductive approach [57], and these data were used to guide the research team in improving the quality of the delivered intervention by informing positive changes in the methodology and design of the intervention for the future implementation in a consequent study.
7. Statistical power and sample size estimation was calculated for meaningful potential future primary outcomes (e.g. loneliness or social support) using a method based on the differences in means between the intervention and control groups, using the G-power software [58].
8. An effect size (ES) was calculated for loneliness, social support, social networks, anxiety and depression, self-efficacy for exercise, satisfaction with level of social contacts, and the expected outcomes and barriers for exercise. Means (M) and standard deviations (SD) were used to investigate the effect size for change in loneliness using mixed between (intervention group vs. control group) and within (over time) repeated-measures analysis of variance (ANOVAs) with post hoc comparisons.

### Data monitoring

The data monitoring committee for this project was the supervisory research team (three academic supervisors). They were responsible for checking accuracy of quantitative data upon assembly of the final database following completion of data collection prior to data analysis. The qualitative data were analysed iteratively by AS with independent analysis and oversight by a member of the supervisory team with expertise in qualitative and mixed methods research (JT). AS was responsible for monitoring and reporting spontaneous adverse events or any unintended trial effects to the supervisory team, and the primary supervisor (AW). The trial was also subject to independent audit request by the sponsor, the University of Birmingham, by a team independent of the supervisory/research team.

### Data collection

Data were collected at the university facility at screening, baseline and post-intervention period (12 weeks after the start of the intervention). After providing baseline eligibility screening, potential participants were offered a total of five visits for health assessments at the university facility. Participants in the intervention group had an additional sixth visit for attending the mid-point focus group.

### Sample size

As this was a feasibility study to inform the design of the future large-scale RCT, a total targeted sample of 40 older adult participants was considered necessary to be recruited for estimation of the critical parameters [33] with 20 in the intervention group and 20 in the WL control group.

### Progression criteria

The progression criteria to a definitive large-scale RCT were the following: (1) no any serious adverse events, such as hospitalisation, life-threatening condition, death and any adverse events associated with the intervention experienced by less than 5% of participants per group; (2) recruitment rate of no less than 75% by the end of the four months recruitment period; and (3) retention rate of no less than 75% in each group at 12 weeks (end-point). If all three criteria were not met, there would be insufficient evidence to justify proceeding to the definitive RCT. No targets were set for other feasibility outcomes, e.g. questionnaire completion rates or attendance at the intervention sessions.

### Data analysis

Quantitative data were analysed using SPSS version 22.0 for Windows (SPSS Inc., Chicago, IL) employing an intention-to-treat analysis (based on their treatment allocation and irrespective of participants’ adherence or withdrawal) [59]. The level of significance was set at *p* < .05; however, any hypothesis testing was preliminary, and any results were interpreted with caution as this feasibility study is underpowered and the analyses based on small numbers. Baseline differences between groups for continuous data (e.g. age, BMI, resting blood pressure, number of comorbidities, cognitive and physical functioning, and outcomes of questionnaires) were analysed using one-way analysis of variance (ANOVA). Chi-squared tests were applied for nominal data (e.g. gender, ethnicity, marital status, living arrangements, level of education, children, and employment status). For descriptive statistics, data were presented as means (M) and standard deviations (SD). Nominal data were presented as number (N) and percentage. Mixed between (group) and within (time) repeated-measures ANOVAs with post hoc comparisons were applied to investigate the effect of the intervention versus control on psychosocial outcomes (loneliness, social support, support networks, depression, anxiety, self-efficacy for exercise, satisfaction with level of social contacts), expected outcomes and barriers for exercise and accelerometer data. The accelerometer data were analysed using the ActivPAL software V7.1.18 (PAL technologies, Scotland, UK). Recorded data were downloaded to a computer, and data for average daily amount of stepping (step counts), average time lying and sitting (h) in increments of 15 s, average time standing (h), and energy expenditure (EE, MET/h) were analysed using mixed between (intervention group) and within (time) ANOVAs. For the Expected Outcomes and Barriers for Habitual Exercise scale [55], additional test-retest reliability was calculated via correlation. In order to explore which outcome measures are likely to be most important for the main trial, Pearson’s correlations were performed between calculated change scores over time in the experimental group for all psychosocial outcomes (Lubben’s social networks, loneliness and self-efficacy for exercise) and change scores for averaged daily physical activity (time lying/sitting (h), time standing (h), time stepping (h), step counts, sit to stand transitions (n) and energy equivalent (METs/h)). Statistical power and sample size estimation for a future large-scale RCT were calculated for meaningful outcomes (e.g. loneliness or social support) using the method based on the differences in means between the intervention and control group using the G-power software Version 3.1 [58].

### Qualitative study

Qualitative data were thematically analysed by two research team members independently using a phenomenological inductive approach [57]. Transcripts were returned to participants for comments/correction to ensure transparency and trustworthiness of the data (member checking) [60]. Validated transcripts were read several times by the two independent researchers to obtain an overall meaning. Then, themes and subthemes with important meanings common across all participants were derived from the obtained data. Results were compared through discussion between reviewers [61]. Data were pseudo-anonymised with a unique identification number (ID) and stored confidentially in locked filing cabinets/on password protected university computers accessible only to the research team. Audio recordings were destroyed after the recordings were transcribed verbatim.

## Results

### Participant characteristics

Twenty-five participants aged 68.5(8.05) years (mean (SD), range 60-92 years) healthy, inactive, community-dwelling older adults, 14 (56%) female, and 18 (72%) white. Baseline descriptive statistics of participants are shown in Table 1.

**Table 1:** Baseline socio-demographic, anthropometric and health-related characteristics of study participants by group (*n* = 25)

Variable	Intervention (***n*** = 12)	Control (***n*** = 13)
Age, years	68.4 (5.9)	67.3 (11.5)
Male, *n* (%)	5 (41.7)	6 (46.2)
Ethnicity, *n* (%)		
White	7 (58.3)	11 (84.6)
Black	2 (16.7)	0 (0)
Asian	1 (8.3)	1 (7.7)
Other	2 (16.7)	1 (7.7)
Marital status, *n* (%)		
Married	4 (33.3)	2 (15.4)
Single/never been married	4 (33.3)	4 (30.8)
Divorced/separated	2 (16.7)	5 (38.5)
Widowed	2 (16.7)	2 (15.3)
Living alone, *n* (%)	8 (66.7)	9 (69.2)
Education, *n* (%)		
No qualification	2 (16.7)	1 (7.7)
Secondary education	2 (16.7)	4 (30.8)
College degree	3 (25)	2 (15.4)
University degree or higher	5 (41.7)	6 (46.2)
Having children, *n* (%)	7 (58.3)	10 (76.9)
Not employed /retired, *n* (%)	10 (83.3)	8 (61.5)
Comorbidities, *n* (%)		
0	3 (25)	3 (23.1)
1	5 (41.7)	2 (15.4)
≥ 2	4 (33.3)	8 (61.5)
Physical function (SPPB score ≥ 9 points)	10.3 (1.2)	10.8 (1.0)
Cognitive function **(**MOCA score ≥ 22 points)	28.3 (1.9)	27.5 (2.4)
Height, m	1.7 (0.1)	1.7 (0.1)
Weight, kg	68.2 (12.8)	68.8 (14.2)
Body mass index, kg/m^2^	24.7 (3.0)	24.7 (3.4)
Systolic blood pressure, mmHg	128.9 (7.9)	133.1 (16.6)
Diastolic blood pressure, mmHg	75.7 (8.9)	77.9 (11.9)

Values are the mean (SD) unless indicated otherwise

*n* number; *SPPB* short physical performance battery, *MOCA* Montreal Cognitive Assessment scale

### Feasibility and safety

#### Recruitment and retention

**Fig. 1:**
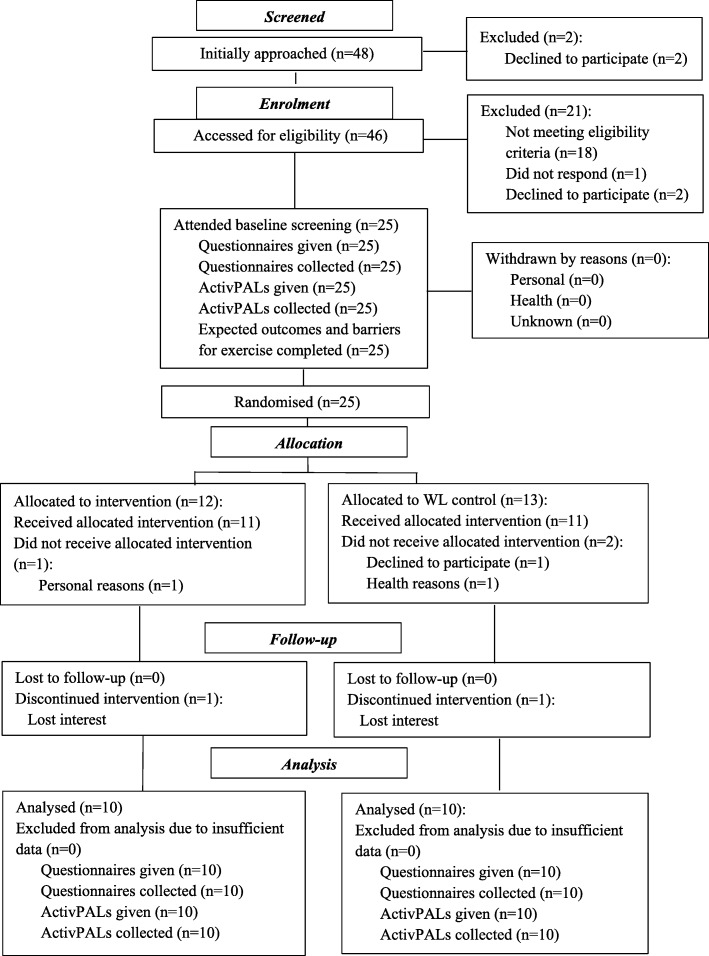
CONSORT flow diagram of Physical Activity Intervention for Loneliness (PAIL) intervention.

The flow of participants through the study is shown in Fig. 1. The intervention was advertised using 192 advertisements (145 leaflets and 47 advertisement posters), which yielded a total of 48 potential participants (45 expressing the initial interest and three recruited through friends’ referrals). The recruitment rate was 25% (48/195). Two declined to participate before completing the phone-based screening. Forty-six potential participants were assessed for eligibility using the phone-based screening with 21 excluded due to not meeting the eligibility criteria (*n* = 18), declined to participate (*n* = 4) and no response (*n* = 1). Reasons for not being eligible were already physically active or taking part in another intervention (*n* = 11), or not at risk of loneliness (as assessed using the phone-screening tool) (*n* = 7). Reasons for declining to participate were pressures of work/lack of time and health reasons. A total of 31 participants (31/46, 67.4%) attended the further eligibility screening at Visit 1, and 25 were eligible to proceed with baseline assessment (Visit 2). Using Wilson’s 95% confidence interval [62], at 12 weeks, 10/12 (83.3%; 95% CI 55.20 to 95.30) intervention and 10/13 (76.9%; 95% CI 49.74 to 91.82) control participants completed final assessments. The retention rate satisfied the criteria of the study, e.g. > 75% of participants at 12 weeks (end-point period), although the recruitment rate of 25% by the end of the 4 months was somewhat lower than was initially proposed at 75%. There were no serious adverse events, or any adverse events observed related to study participation.

#### Attendance

The average attendance rate for the total of 12 sessions of the walking intervention was 58.3% for the intervention group, with attendance ranging from 33.0% to 75.0% (Additional file 6). The average attendance rate for the WL control group was 42.3%, with attendance ranging from 23.1 to 69.2 %. The mean (SD) number of attended sessions per person was 8.6 (2.8) and 6.6 (2.6) in the intervention and wait-list control group, respectively. After completing the 12-week intervention, 7/10 participants from the intervention group and 6/10 participants from the wait-list control group continued walking. The follow-up attendance rate assessed during the 12-week post-intervention period was 48% for the intervention and 52% for the WL control group.

#### The assessment rate of questionnaires

Baseline questionnaires and accelerometer data were provided by 100% (25/25) of participants recruited into the study. Post-intervention questionnaires and end-point accelerometer data were provided by 100% (10/10) of the intervention and 100% (10/10) WL control group participants.

#### The appropriateness of the assessment tools

The average reliability was high for all psychosocial outcomes: loneliness (Cronbach’s alpha 0.857), social support (Cronbach’s alpha 0.975), Lubben’s social networks (Cronbach’s alpha 0.721), depression (Cronbach’s alpha 0.744), anxiety (Cronbach’s alpha 0.693) and self-efficacy for exercise (Cronbach’s alpha 0.925). The expected outcomes for the exercise sub-scale of the Expected Outcomes and Barriers for Exercise questionnaire showed high internal consistency reliability at baseline, with Cronbach’s alpha equalling 0.926 (a week before) and 0.938 (a week after); at post-intervention the value was 0.976. Barriers for the exercise sub-scale of the Expected Outcomes and Barriers for Exercise questionnaire showed high internal consistency reliability at baseline, with Cronbach’s alpha equalling 0.888 (a week before) and 0.924 (a week after); at the post-intervention period the value was 0.943.

### Findings from the qualitative study

#### The appropriateness, practicality and acceptability of the intervention by participants

A total of 5/12 (42%) participants in the intervention group attended two focus group interviews, at the mid-point and end-point intervention periods. The focus group attendees were representative of the overall intervention group characteristics. The responses of participants during the focus groups were summarised in the main themes for mid-point and end-point intervention periods and presented below.

## 1. Mid-point focus groups

Participants ranged in age from 62-76 years, 60% (3/5) female, 80% white, with 60% (3/5) living alone, having a university degree, having children, being retired and having one medical condition. The main themes were study design and recruitment (3 subthemes), healthy workshops (3 subthemes) and walking sessions (3 subthemes) (Additional file 4).

Mid-point focus group results showed that in terms of the appropriateness and practicality, the spring-summer seasons were a better to start the intervention than winter time. Overall, participants had very positive views of the walking intervention, particularly the benefits of walking, its cost-effectiveness in terms of the economic benefits for older adults and direct positive health effects:

And because I am on a fixed income now, you know I can’t just go out and earn a bit more money to do something, it does limit you a little bit in what you can do and you have got all this time, but you haven’t got the money. Err, you know, and I brought up a child on my own so she took quite a lot of my salary when I was working [Kate laughs], you know I have never got a lot of money (all laugh) to do what I would really like to do. So, you have to work within that (.) (Alison, 75, female).

For others, it was a chance to meet new people and get access to local community groups:

I mean walking is good because it loosens everybody up a bit, gets people to know each other (.) (Andrew, 68, male).

The content of the healthy workshops was relevant and allowed them to share feelings and knowledge. However, the main barriers to attend walks were (1) personal, such as lack of time associated with family and community celebrations, holiday, home refurbishments, and carer responsibilities; and (2) environmental barriers, such as transportation and the weather (Additional file 3). Participants found it difficult to get to the location by public transport, or to find the nearest parking area if the meeting point was on campus:

And some of the walks, like [the place of the walk], although it wasn’t that particularly early, it’s getting there (.) [an issue] on public transport (Sarah, 76, female).

## 2. End-point focus groups

Participants ranged in age from 65-76 years, 20% (1/5) female, 80% white, 60% (3/5) living alone, 40% (2/5) with university degree, 60% having children, retired and having one or more medical condition. The main themes were study design and recruitment (3 subthemes), healthy workshops (3 subthemes), and walking sessions (2 subthemes) (Additional file 5). Participants felt that participation in the intervention helped them to become more physically active, which was their initial aim:

You have to just try and keep motivating [yourself], just keep going I suppose, rather than just sitting at home. I don’t know how (.) for me like, I am working four days a week at the moment, so I don’t know how I would feel when I retire, which is going to happen next year so (.)” (Ben, male, 65).

“I enjoyed the exercise thing. It is quite, you know (.) it is just (.) She (referring to the exercise leader) sort of said, you know, made us aware of sort, of what sort of exercise is good for, what parts of your body and so forth. So, I mean it is important, isn’t it to keep moving, keep active and this is what part of the programme is about, isn’t it? (Ben, male, 65).

Common interests raised during the walks allowed first friendship gains that started from as early as the second or third walk and continued after the programme’s end at 12 weeks. Walking was seen to promote the bonding of participants and improved their aspirations for friendship-based relationships:

I have definitely made new friends, enjoyed meeting new people and you gel with some people which is a human nature, so (.) (Sarah, female, 76).

Future recommendations included more group leaders per group and classification of walks by ability level (e.g. beginner, improver):

(.) and it was only (the Researcher). But if (the Researcher) had two other people on our walk (.). You can do a slow one slightly less (distance), a medium one slightly further and a faster one even further. I don’t know how you would organise that, but that would take care of the pacing (.) The Researcher will need help. You can’t do that with one person because you got to lead, so she actually does need somebody to lead a group (Alison, 75, female).

Another suggestion was to conduct healthy workshops during a separate session/time:

“-You suggest a separate session for loneliness workshops? (Focus group lead).

-A separate [Kate: a separate session yeah (.)] without so many leaflets (Alison, 75, female).

-Yeah, so that will be a little bit (.) it will be a good focus for each of us to learn, to share (.) (Kate, 62, female).

### Changes in outcome measures

There were no significant differences between the intervention and control groups at baseline in all measures except for number (*n*) of sit-to-stand transitions, which were 14.4 points lower in the intervention group (mean 43.3(11.3)) compared with controls (mean 57.6(15.8); 95% CI 2.91, 25.81). Table 2 shows the between group differences for secondary outcomes. In general, a pattern of improvement was seen across all psychosocial and physical activity outcomes in the intervention group. All correlations performed for psychosocial outcomes are shown in Table 3. Correlation analysis performed for calculated change scores over time in the experimental group for all psychosocial outcomes showed no significant correlations between any other psychosocial outcomes except for a moderate negative correlation between self-efficacy for exercise and loneliness, and a moderate negative correlation between self-efficacy for exercise and the family sub-scale of Lubben’s social networks. A moderate positive correlation emerged between self-efficacy for exercise and the friendship sub-scale of Lubben’s social networks, such that an increase in self-efficacy for exercise was associated with a larger family and friends social network size.

**Table 2:** 12-week group differences between intervention and control groups in anthropometric, health-related, physical activity and psychosocial outcomes from baseline

Variables	Mean (SD)		Difference (95% CI)
	Intervention (***n*** = 12)	Control (***n*** = 13)	
Height, m	1.7 (0.1)	1.7 (0.1)	− 0.0 (− 0.1 to 0.1)
Weight, kg	67.9 (12.6)	68.4 (14.2)	− 0.5 (− 11.6 to 10.7)
BMI, kg.m^-2^	24.3 (2.8)	24.5 (3.5)	− 0.2 (− 2.9 to 2.4)
SBP, mmHg	123.3 (8.3)	129.9 (13.8)	− 6.6 (− 16.1 to 2.9)
DBP, mmHg	74.1 (8.9)	74.5 (9.6)	− 0.4 (− 8.1 to 7.3)
Loneliness	18.1 (5.2)	18.6 (5.2)	− 0.5 (− 4.8 to 3.8)
Social support	63.9 (19.8)	59.8 (20.7)	4.1 (− 12.7 to 20.9)
LSN (total)	15.4 (5.0)	12.0 (6.3)	3.4 (− 1.3 to 8.2)
LSN (family)	6.9 (4.4)	5.9 (3.8)	1.0 (− 2.4 to 4.4)
LSN (friends)	8.5 (2.5)	6.1 (4.9)	2.4 (− 0.9 to 5.7)
Depression	6.5 (3.0)	5.5 (3.8)	1.0 (− 1.8 to 3.9)
Anxiety	6.9 (3.3)	7.4 (3.7)	− 0.5 (− 3.4 to 2.5)
SEE	7.1 (1.7)	5.2 (2.2)	1.9 (0.3 to 3.6)
SSC	6.5 (3.1)	5.3 (3.6)	1.2 (− 1.7 to 4.1)
Expected outcomes	3.8 (1.1)	3.9 (0.8)	− 0.1 (− 0.9 to 0.7)
Barriers for exercise	2.6 (0.9)	2.9 (1.0)	− 0.3 (− 1.0 to 0.5)
Time lying/sitting (h)	16.8 (1.9)	17.1 (1.8)	− 0.3 (− 1.8 to 1.2)
Time standing (h)	5.5 (1.5)	5.1 (1.6)	0.5 (− 0.9 to 1.7)
Time stepping (h)	1.9 (0.7)	1.8 (0.7)	0.1 (− 0.6 to 0.7)
Step counts	9067.5 (4355.7)	8575.6 (4117.5)	491.9 (− 3013.6 to 3997.5)
Sit to stand transitions (n)	45.3 (10.6)	60.3 (14.6)	− 15.0 (− 25.7 to − 4.4)
Energy Equivalent (METs/h)	34.4 (1.7)	34.1 (1.6)	0.3 (− 1.1 to 1.7)

*BMI* body mass index, *DBP* diastolic blood pressure, *n* number, *SBP* systolic blood pressure, *SEE* self-efficacy for exercise; *SSC* satisfaction with social contacts, *LSN* Lubben’s social networks

**Table 3:** Pearson product-moment correlations between change scores of psychosocial outcomes in the Intervention group (*n* = 12)

Scale	1	2	3	4	5	6	7	8	9	10
1. UCLA-8	-									
2. MOSSSS	0.046	-								
3. LSN (total)	− 0.140	0.107	-							
4. LSN (family)	0.278	0.044	0.593*	-						
5. LSN (friends)	− 0.470	0.057	0.313	− 0.579*	-					
6. Depression	0.411	− 0.506	0.315	0.126	0.171	-				
7. Anxiety	0.183	− 0.503	− 0.161	− 0.238	0.117	0.530	-			
8. SEE	− 0.707*	− 0.148	− 0.108	− 0.648*	0.655*	− 0.197	0.131	-		
9. SSC	− 0.597	0.381	0.261	0.050	0.216	− 0.320	− 0.741**	0.425	-	
10. Expected outcomes	− 0.043	0.209	0.332	0.223	0.073	− 0.106	− 0.722**	− 0.240	0.563	-
11. Barriers for exercise	− 0.229	− 0.208	− 0.205	− 0.437	0.309	0.133	− 0.033	0.414	0.422	− 0.102

*UCLA-8* 8-item University of California at Los Angeles loneliness scale; *MOSSSS* medical outcomes study social support survey, *LSN* Lubben’s social networks, *SEE* self-efficacy for exercise, *SSC* satisfaction with level of social contacts

*Significant correlation at *p* < 0.05 (two-tailed)

**Significant correlation at *p* < 0.01 (two-tailed)

### Power calculation

The potential sample size for a future large-scale trial was calculated for each psychosocial outcome using post hoc analyses first to estimate the observed power based on the effect sizes from the repeated measures between-within ANOVAs on the 25 participants using the partial eta-squared from the interaction effect (*η*^2^). Following this, a sample size was calculated a priori for a future trial using *α* = 0.05 and power at 80% for each measure: loneliness (*n* = 72, *η*^2^ = 0.014), social support (*n* = 48, *η*^2^ = 0.030), Lubben’s social network (*n* = 48, *η*^2^ = 0.026), depression (*n* = 378, *η*^2^ = 0.008), anxiety (*n* = 68, *η*^2^ = 0.032), SEE (*n* = 12, *η*^2^ = 0.122), expected outcomes (*n* = 60, *η*^2^ = 0.033) and barriers for exercise (*n* = 172, η^2^ = 0.011) (Additional file 7). The calculation of estimated sample size for SSC was not possible as *η*^2^ = 0.000 (insufficient power).

## Discussion

This study assessed the feasibility of the Physical Activity Intervention for Loneliness (PAIL) intervention in community-dwelling older adults at risk for loneliness. Based on the progression criteria, the retention rate was satisfactory, e.g. > 75% of participants at 12 weeks (end-point period), as well as no adverse events during the intervention. The recruitment rate of 25% by the end of the 4 months was somewhat lower than initially proposed at 75%. Therefore, only two out of three criteria of progression to the definitive RCT were satisfied, meaning that the study was not feasible to deliver in its present form. However, these findings were not surprising based on the inability to accurately estimate recruitment rates in the present study, as well as the fact that it is difficult to access socially isolated older adults who may be less interested in joining an intervention than those who are more socially engaged. Therefore, recruitment from GPs may be more advantageous than advertisement via mass media resources such as leaflets or advertisement posters in a future large-scale trial to recruit older adults at high risk of loneliness or social isolation [20, 63, 64].

The low attendance rate (58.3% for the intervention group, 42.3% in the WL control group) in this study is not surprising given that the PA intervention is considered to be a behaviour change strategy that is not easily initiated or consistently maintained in older adult populations [65]. Based on participant responses, providing transport to and from walking session locations may significantly improve adherence and provide easier access to various locations of walks to maintain interest of older adults.

No significant changes in outcome measures were found after 12 weeks of the PAIL intervention. As reported in the literature, the acute exercise effect is brief [66] and a longer duration intervention as well as an adequate follow-up period of the future intervention may be needed to allow participants to build upon transforming new contacts into meaningful relationships based on trust, which previous studies suggest may be up to 5 months [67, 68].

Given that the initial aim of the intervention was to see if loneliness could be impacted, and the observed power and estimated sample size for this seems achievable, this could be recommended as a future primary outcome. However, a feasibility study, by its very nature, may be under powered to achieve statistical significance at *α* = 0.05 [69]. Therefore, any interpretation based on significance levels should be treated with caution. Post hoc sample size calculations were possible; however, are not advisable for feasibility studies [70]. Therefore, additional feasibility testing is recommended using the minimum clinically important difference (MCID) [69], which was performed for depression and anxiety as they had a set cut-off point of 4 scores. The mean between group difference at 12 weeks was non-significant and less than the a priori determined MCID of 4 points with 95% CI crossing zero (MD = 1.0, 95% CI: − 1.8 to 3.9, *p* = 0.457), suggesting that the results are equivocal. Similar results were obtained for anxiety (MD = − 0.5, 95% CI, − 3.4 to 2.5, *p* = 0.744). Given the small effect sizes for SCC, a sample size calculation was not possible, thus future feasibility testing of this measure is advised. The efficacy outcomes of the current feasibility study may be used in exploratory analyses, but further changes in the intervention design and methods are required before proceeding to a definitive trial. For example, a larger sample and more rigorous recruitment strategy, as well as easier to access walking locations may significantly improve the quality of future research. A future intervention would also be advised to (1) classify walking groups by ability level; (2) add more group leaders per group; (3) conduct healthy workshops during a separate session/time; (4) provide transport to walking locations in order to maintain high adherence and diversity of routes; and (5) conduct focus group discussions for control participants to understand their experience of the research processes, questionnaires and other elements.

### Strengths and limitations

This study had a robust design and highlighted the importance of PA interventions for loneliness in older adults based on the lack of existing research [20]. Walking was chosen as it has been shown to be the most feasible and cost-effective method of physical activity for older adults [71, 72]. Other strengths of this study include objective measurement of PA, use of reliable methods of assessment of psychosocial outcomes in older adults, and the mixed methods research design that allowed for collecting feedback from participants during and at the end of the intervention.

Study limitations include selection bias associated with the recruitment of physically mobile participants as assessed during the eligibility screening. Therefore, any treatment effect of this feasibility study may be blunted by this selection bias [14, 21] and inclusion of higher functioning older adults. The identification of sedentary individuals in this study was done using the modified short form of the CHAMPS physical activity questionnaire adopted for use in an older adult population [73]. For future studies, it is advisable that instead of using this general normative definition of a sedentary individual, this exclusion criterion could be exclusive to walking. Future studies should consider using objective methods of assessment of PA (e.g. pedometers or accelerometers) in older adults in addition to the phone-based screening for a rigorous eligibility process. The optimum dose was not a feasibility outcome in the present study. However, the low attendance suggested that more frequent sessions would not be feasible. In terms of PA, the ideal dose would be a total of 150 min per week, but the present study suggests this is unlikely. Appropriate blinding of the researcher was not possible in the present study due to a lack of resources available to pay an independent person to deliver the intervention and collect all of the data. As such, it is recommended that future studies are resourced to allow for the recruitment of a trained walking leader to deliver the intervention and an independent assessor of outcomes to allow for adequate blinding and reduce detection bias. The mixed design of the intervention allowed for the enrichment of the quantitative data of the intervention by including the opinion of participants about the study. However, the low attendance of focus groups was a limitation. Out of 12 people in the intervention group, only 58.3% attended the 12-week intervention with a mean (SD) number of attended sessions per person 8.6 (2.8). The reasons for not attending focus groups were work/lack of time, health reasons and other (e.g. transport difficulties, lack of motivation, family reasons). In addition, older adults with loneliness may have barriers for open discussions due to the “stigmatising nature of loneliness,” [14, 74–76]. Therefore, a future study may consider using one-to-one interviews instead. In addition, WL control group participants may have experienced a significant nocebo effect (a worsening symptom or disappointment) [77]. Compared to control group designs with no treatment, participants with WL control group designs have a hope for the intervention delivered later; however, this may also induce frustration [78]. This may be especially true for lonely older adults with higher levels of depression or anxiety, and the likelihood of worsening their psychological well-being is high which, in turn, may influence questionnaire responses. Future research should attempt to address these issues by changing the control group design to a no treatment control, but this brings its own ethical issues surrounding not offering an intervention to individuals who have the potential to benefit from it. It should be acknowledged that even with the use of the loneliness screening measure, individuals with highest loneliness risk may be those least likely to respond to an invitation to eligibility screening due to low confidence and motivation.

## Conclusions

The present study suggests that community-dwelling older adults at risk for loneliness can successfully complete a 12-week walking intervention programme, reporting enjoyment and benefits, and they were keen to share their knowledge and experiences during the healthy/social workshops. The efficacy outcomes of the current feasibility study may be used in exploratory analyses, but the changes suggested above to the intervention design and methods would be necessary before proceeding to a definitive trial. Further feasibility testing based on the different CIs with a MCID set a priori would be advisable.

## Supplementary information

**Additional file 1.** CONSORT extension to pilot and feasibility trials checklist

**Additional file 2.** The TIDieR checklist

**Additional file 3.** Walking programme

**Additional file 4.** Main themes and emerged sub-themes from the mid-point focus group interviews

**Additional file 5.** Main themes and emerged sub-themes from the end-point focus group interviews

**Additional file 6.** Weekly average attendance rate (%) for 12 weeks walking intervention

**Additional file 7.** Sample size calculations for psychosocial outcomes

## Data Availability

Not applicable.

## Abbreviations

ANOVA: Analysis of variance
β: The standardised regression coefficient
BMI: Body mass index
BP rest: Resting blood pressure
CHAMPS: Community Healthy Activities Model Programme for Seniors
CI: Confidence intervals
EE: Energy expenditure
ES: Effect size
GP: General practitioner
HADS: Hospital anxiety and depression scale
LSNS-6: 6-item Lubben’s Social Network Scale
M: Mean
MCID: Minimal clinically important difference
MET: The metabolic equivalent
MOSSSS: Medical Outcomes Study Social Support Survey
MOCA: Montreal Cognitive Assessment
MVPA: Moderate-to-vigorous physical activity
N: Number
NHS: National Health Services
PA: Physical activity
PAIL: Physical activity intervention for loneliness
R: Correlation coefficient
RCT: Randomised controlled trial
RPE: Ratings of perceived exertion scale
SD: Standard deviation
SEE: Self-efficacy for exercise scale
SSC: Satisfaction with level of social contacts
SPPB: Short physical performance battery
STEM Ethical Review Committee: Science, Technology, Engineering and Mathematics Ethical Review Committee
TIDieR: Template for intervention description and replication checklist
UCLA-8: 8-item The University of California, Los Angeles Loneliness Scale
UK: United Kingdom
WL: Wait-listed control group
