# Supplementary material for: Physical Activity Intervention for Loneliness (PAIL) in community-dwelling older adults: a randomised feasibility study
Source: Pilot Feasibility Stud. 2020 May 23;6:73. doi: 10.1186/s40814-020-00587-0 (PMC7245022; doi:10.1186/s40814-020-00587-0)
Supplement: Supplementary file 1 — Additional file 1. CONSORT extension to pilot and feasibility trials checklist [file 40814_2020_587_MOESM1_ESM.docx]

**Additional file 1** CONSORT extension to pilot and feasibility trials checklist

| **Section/topic and item number** | **Checklist item** | **Page** |
| --- | --- | --- |
| ***Title and abstract*** |  |  |
| 1a | Identification as a pilot or feasibility randomised trial in the title | 1 |
| 1b | Structured summary of pilot trial design, methods, results, and conclusions | 1-2 |
| ***Introduction*** |  |  |
| 2a | Scientific background and explanation of rationale for future definitive trial and reasons for a randomised feasibility trial | 2-5 |
| 2b | Specific objectives or research questions for a feasibility trial | 6 |
| ***Methods*** |  |  |
| Trial designs: |  |  |
| 3a | Description of feasibility trial design including allocation ratio | 6 |
| 3b | Important changes to methods after feasibility trial commencement (such as eligibility criteria), with reasons | N/A |
| Participants: |  |  |
| 4a | Eligibility criteria for participants | 8 |
| 4b | Settings and locations where the data were collected | 7 |
| 4c | How participants were identified and consented | 7 |
| Interventions: |  |  |
| 5 | The interventions for each group with sufficient details to allow replication, including how and when they were actually administered | 8-9 |
| Outcomes: |  |  |
| 6a | Completely defined prespecified assessments or measurements to address each pilot trial objective specified in 2b, including how and whet they were assessed | 10 |
| 6b | Any changes to pilot trial assessments or measurements after the feasibility trial commenced, with reasons | N/A |
| Sample size: |  |  |
| 7a | Rationale for numbers in the feasibility trial | 12-13 |
| 7b | When applicable, explanation of any interim analyses and stopping guidelines | N/A |
| Randomisation: |  |  |
| Sequence generation: |  |  |
| 8a | Method used to generate the random allocation concealment | 7 |
| 8b | Type of randomisation; details of any restrictions. | 7 |
| Allocation concealment and mechanism |  |  |
| 9 | Mechanism used to implement the random allocation sequence (such as sequentially numbered containers), describing any steps taken to conceal the sequence until interventions were assigned | 7 |
| Implementation |  |  |
| 10 | Who generated the random allocation sequence, enrolled participants, and assigned participants to interventions | 7 |
| Blinding: |  |  |
| 11a | If done, who was blinded after assignment to interventions (e.g. participants, care providers, those assessing outcomes and how). | 7 |
| 11b | If relevant, description of the similarity of interventions | N/A |
| Analytical methods: |  |  |
| 12 | Methods used to address each feasibility trial objective whether qualitative or quantitative | 13 |
| ***Results*** |  |  |
| Participant flow (a diagram is strongly recommended) |  |  |
| 13a | For each group, the numbers of participants who were approached and/or assessed for eligibility, randomly assigned, received intended treatment, and were assessed for each objective | 16 |
| 13b | For each group, losses and exclusions after randomisation, together with reasons | 17 |
| Recruitment: |  |  |
| 14a | Dates defining the periods of recruitment and follow-up | 7 |
| 14b | Why the pilot trial ended or was stopped | 15 |
| Baseline data: |  |  |
| 15 | A table showing baseline demographic and clinical characteristics for each group | Table 2 |
| Numbers analysed: |  |  |
| 16 | For each objective, number of participants (denominator) included in each analysis. If relevant, these numbers should be by randomised group | 15-17 |
| Outcomes and estimation: |  |  |
| 17a | For each objective, results including expressions of uncertainly (such as 95% confidence interval) for any estimates. If relevant, these results should be by randomised group | 15-17 |
| Ancillary analyses: |  |  |
| 18 | Results of any other analyses performed that could be used to inform the future definitive trial | 19-20 |
| Harms: |  |  |
| 19 | All important harms in each group | N/A |
| ***Discussion*** |  |  |
| Limitations: |  |  |
| 20 | Feasibility trial limitations, addressing sources of potential bias and remaining uncertainly about feasibility | 20-21 |
| Generalisability: |  |  |
| 21 | Generalisability (applicability) of feasibility trial methods and findings to future definitive trial and other studies | 21-22 |
| Interpretation: |  |  |
| 22 | Interpretation consistent with feasibility objectives and findings, balancing potential benefits and harms, and considering other relevant evidence | 22 |
| 22a | Implications from progression from feasibility to future definitive trial, including any proposed amendments | 22 |
| ***Other information*** |  |  |
| Registration: |  |  |
| 23 | Registration number | 2 |
| Protocol: |  |  |
| 24 | Where the pilot trial protocol can be assessed, if available | 2 |
| Funding: |  |  |
| 25 | Sources of funding and other support | 23-24 |
| 26 | Ethical approval or approval by research review committee, confirmed with reference number | 6 |
