# Supplementary material for: Physical Activity Intervention for Loneliness (PAIL) in community-dwelling older adults: a randomised feasibility study
Source: Pilot Feasibility Stud. 2020 May 23;6:73. doi: 10.1186/s40814-020-00587-0 (PMC7245022; doi:10.1186/s40814-020-00587-0)
Supplement: Supplementary file 3 — Additional file 3. Walking programme [file 40814_2020_587_MOESM3_ESM.docx]

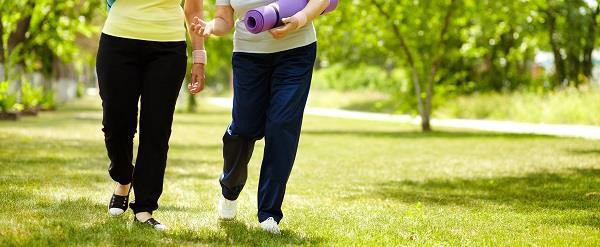


Walking for adults programme


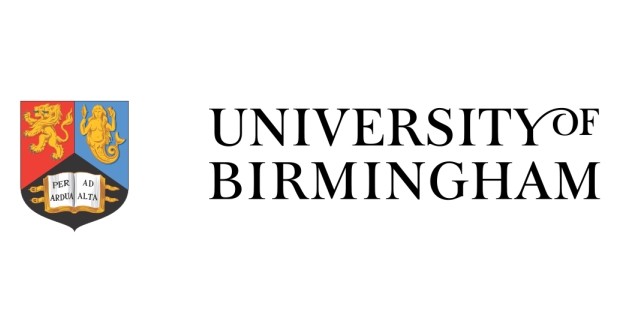


**What are benefits of walking?**


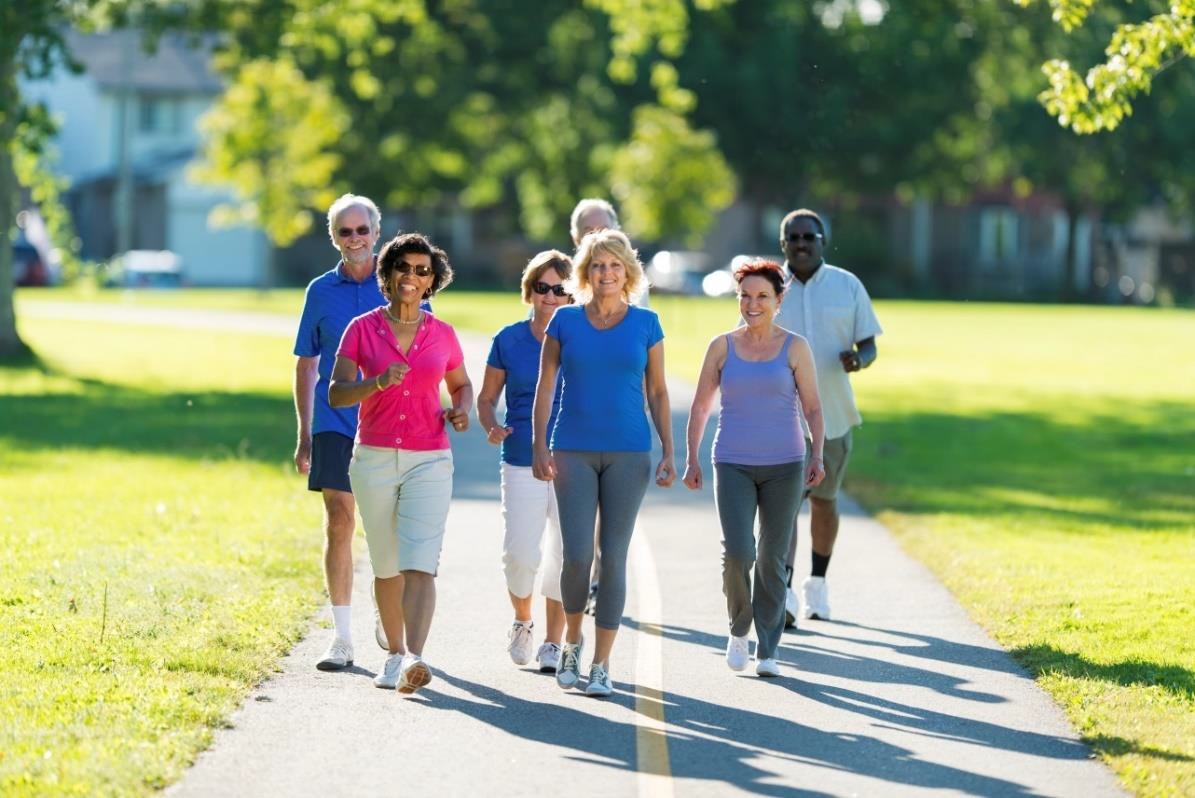


**Live longer and stronger:** boost immunity, keep your weight in check,

reduced risk of heart disease

**Lowers blood pressure**

**Improve mental health:** less depression & anxiety

**Feel better:** good sleep, look happier, boosted mood and self-esteem

**Weekly walks**

1. Saturday 9th of June 10.00-11.00 - **Vale & coffee at Winterbourne gardens**

2. Saturday 16th of June 10.00-11.00 - **Mac, The Cannon Hill Park & Picnic**

3. Saturday 30h of June 10.00-11.00 - **Warley Woods Park**

**3*** Sunday 1st of July 12.00-14.00 - **Richmond Park & Isabella Plantation (London)**

4. Thursday 5h of July 10.30-11.30 **– Lickey Hills Country Park**

**Focus group interview Wed 11th of July at 11.30-12.30**

5. Saturday 14h of July 10.00-11.00 **– Vale & Winterbourne gardens**

6. Saturday 21st of July 10.00-11.00 – **Woodgate valley country park**

7. Wednesday 25th of July 11.00-12.00 - **Vale & coffee at Winterbourne gardens**

**7*** Sunday 29th of July 12.00-14.00 **- Bushy park & Waterfall gardens (London)**

8. Wednesday 1st of August 11.00-13.00 – **Metchley park, Groove Park & Harborne**


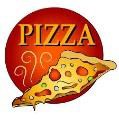


9. Wednesday 8th of August 11.00-12.00 – **Cannon Hill Park**

**Focus group interview Tuesday 14th of August at 13.15-14.15**

10**.** Wednesday 15th of August 11.00-12.00 - **Walk to the Vale (Birmingham)**

11. Friday 24th of August 11.30-12.30 – **Sandwell Valley Park and Forge Mill lake**

12. Wednesday 29th of August 11.00-12.00 - **Vale & coffee at Winterbourne gardens**

**How can I take part?**


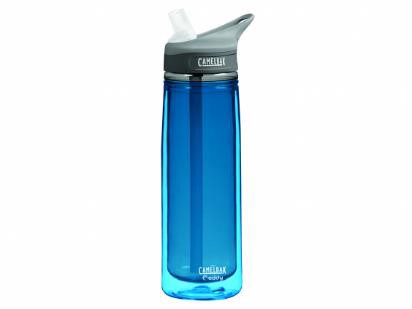


• Just turn up 15 minutes before walk at the starting point and check in with the walking leader.

• No special equipment needed – just wear comfortable clothes and footwear. Bring some water with you.

• All walks returned to the start point unless stated otherwise.

• Due to changes in walking conditions some

walks can be cancelled.

**Have you filled pre-participation**


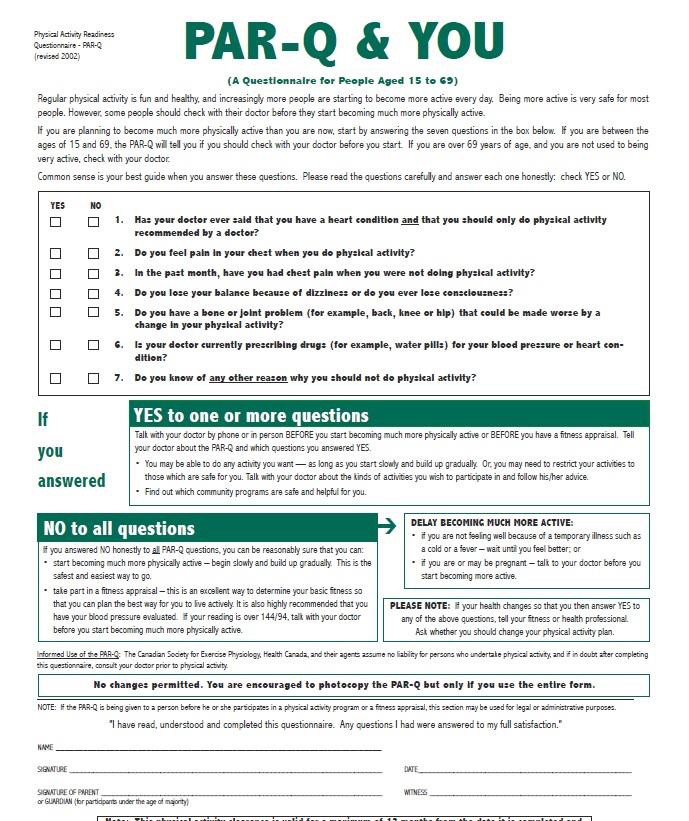


**form?**

• There are minimal risks

in taking part in the PAIL

study. During the

guided walking of a

moderate intensity you

may feel tired or out of

breath, but this is

normal and safe.

Download the Polar beat application on the phone to

measure you heart rate.


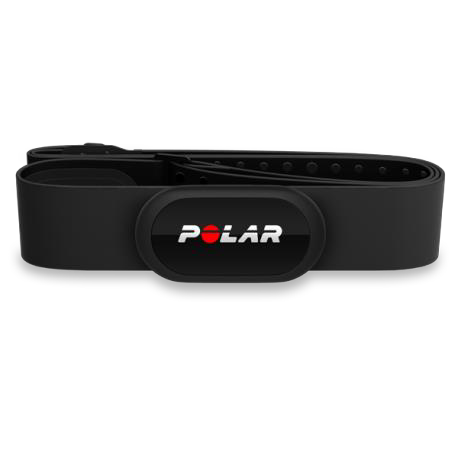

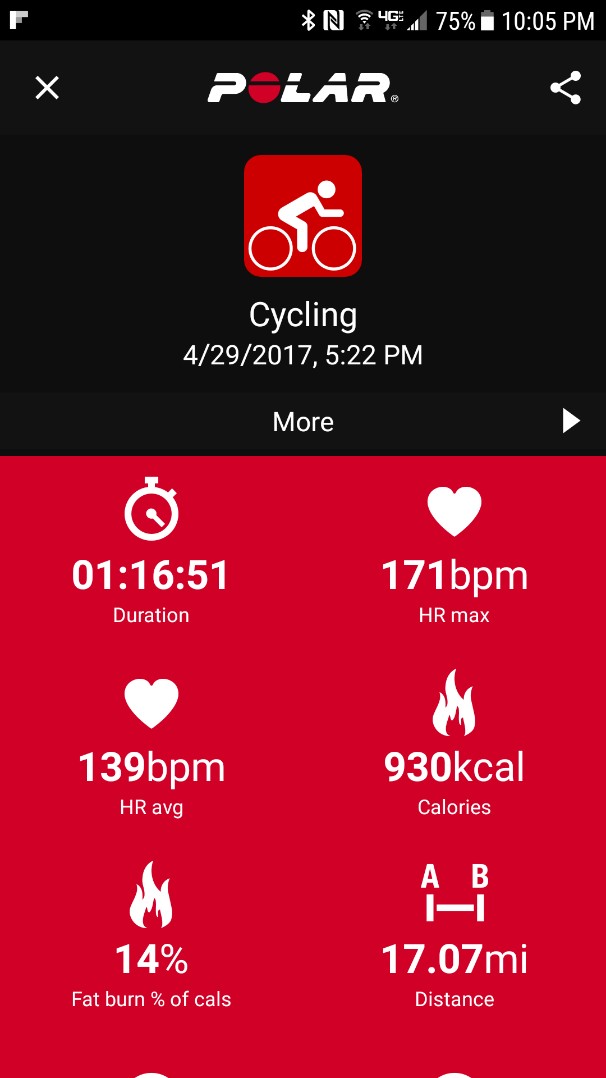


Ask for Polar chest strap to measure HR and a distance

**Download Polar Beat on the Apple store:**

<https://itunes.apple.com/us/app/polar-beat/id555252645?mt=8>

**What if I would like to withdraw?**

• The participation is entirely voluntary

• Everyone can withdraw from the study at any time point without giving a reason

• Any feedback will be valuable

**Parking**

• The University has two main pay and display car parks:

• **North East Car Park** (access via Pritchatts Road). Sat Nav

Postcode: B15 2SA

*Please note that this car park is multistory and does not*

*have a lift.*

**Pritchatts Road Car Park** (at junction with Vincent Drive).

Sat Nav Postcode: B15 2QU

• Free parking for Blue Badge holders is available on campus.

Please drive up to the security barriers at East Gate (access

off Edgbaston park Road, near to the Barber Institute of

Fine Arts) for assistance.

**Week 1- Vale & Winterbourne House**

**and Gardens**


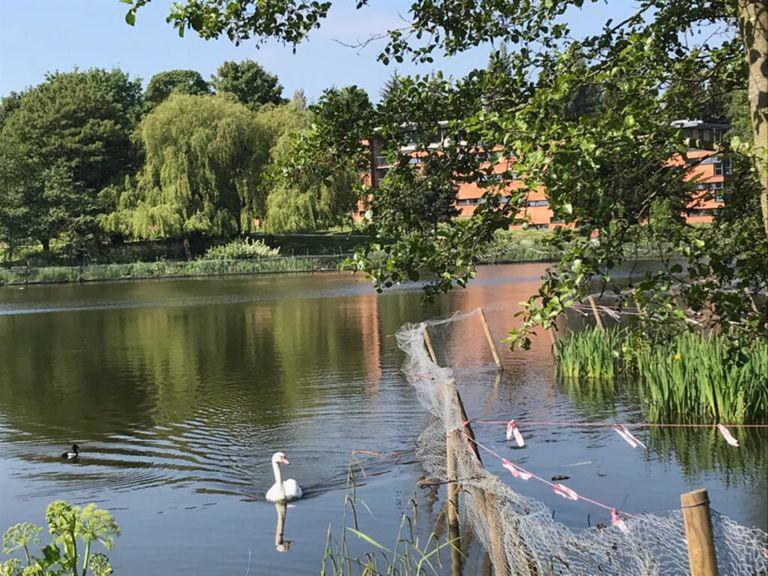


• Saturday 10.00am

• 9thth of June,

• Start and endpoint: University of Birmingham train station

• Walk along the canal up to the Vale

• Getting there: All bus and train routes to the

University train station

• Parking available free of charge near University train station. Use car pooling due to limited places if you are parking at Winterborne House and Gardens (not free of charge).

* *Coffee stop at Winterbourne Gardens (free entry)*

**Vale & Winterbourne House**

**and Gardens**


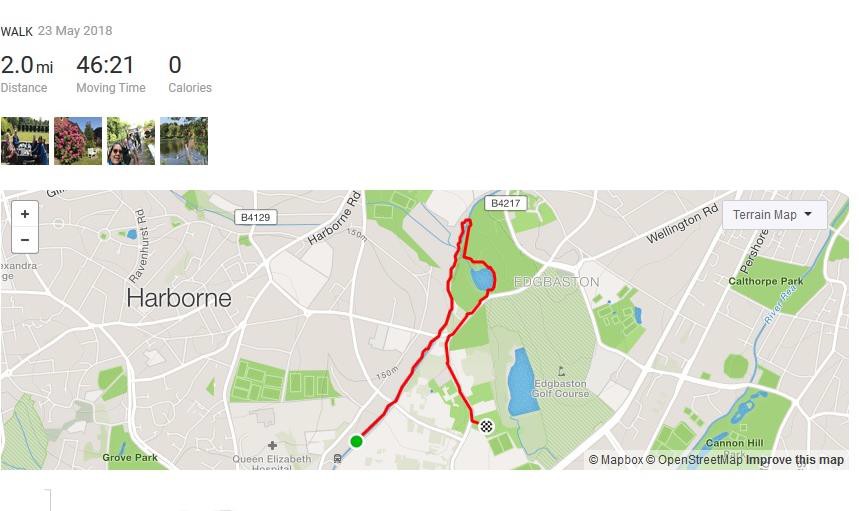


**Topic for discussion**


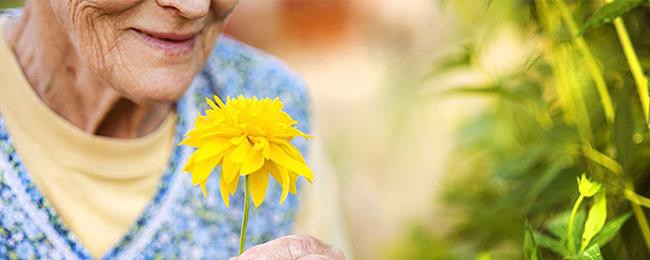

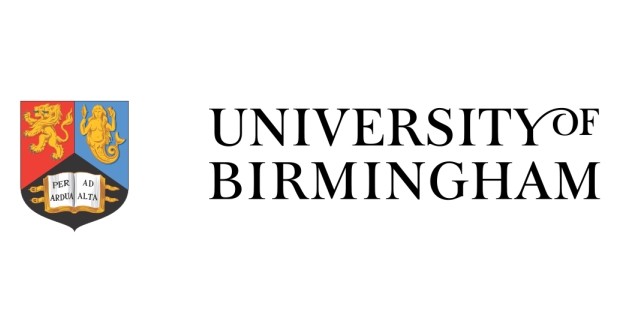


**Healthy ageing**

Wale and Winterbourne House and

Gardens.. Coffee stop


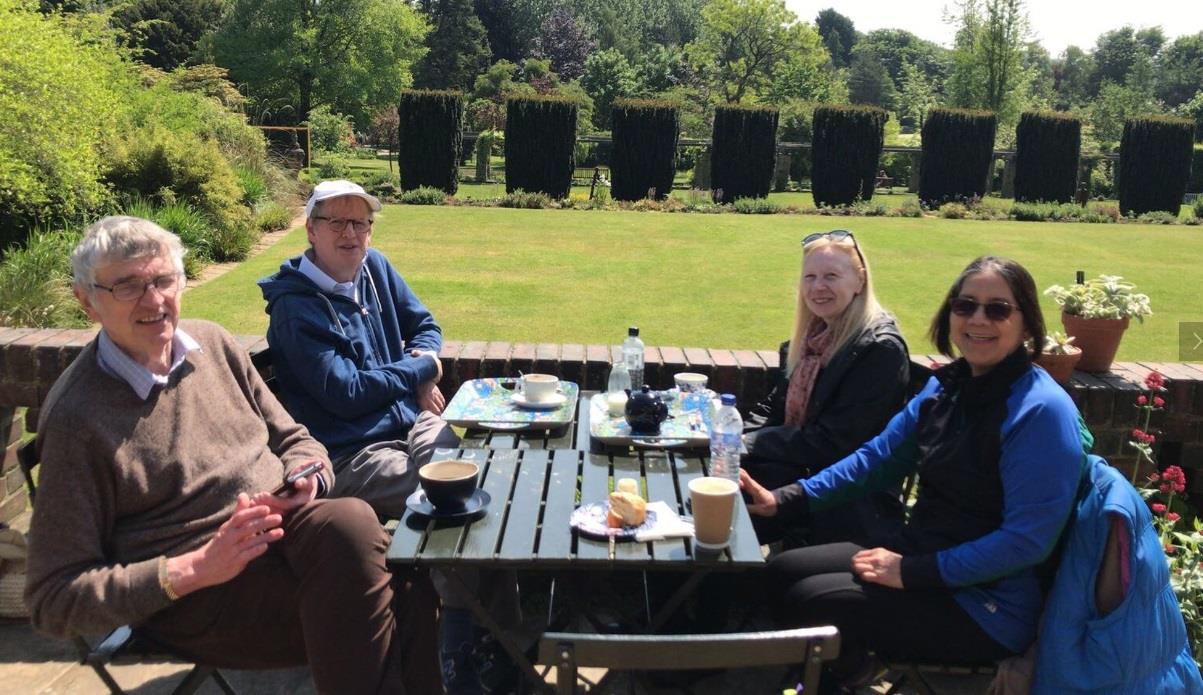


**Week 2 - Cannon Hill park**


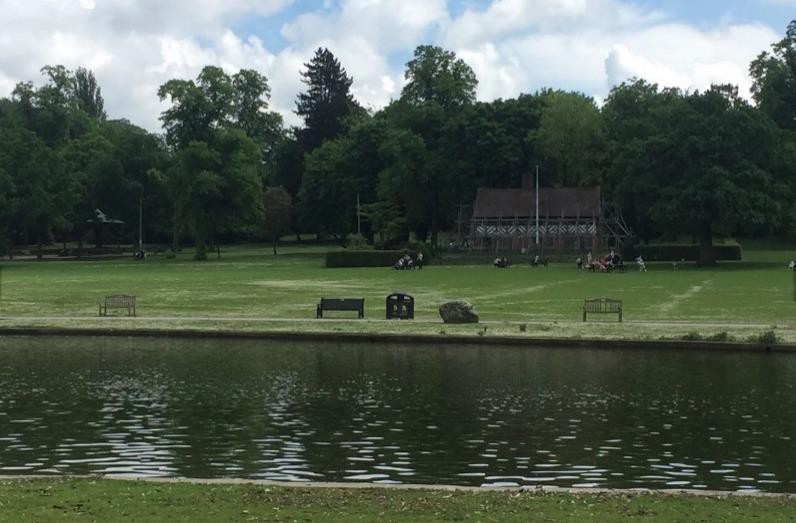


• Saturday 10.00am

• 16th of June,

• Start and endpoint:

MAC, the Cannon Hill Park

• Getting there: From Selly Oak – bus No63 (Bristol

Road/Dawlish road) to Bristol Road/Priory Road.

• Bus X61, 144 from Bristol Road.

• Parking: There are two car parks, one by the MAC off Edgbaston Road £2 up to 4 hours, and a smaller car park off the Russell Road.

• *Coffee stop the Mac café.*

**Mac, Cannon Hill park**

**Mac,** Cannon Hill Park Queen's Ride, Birmingham B12 9QH


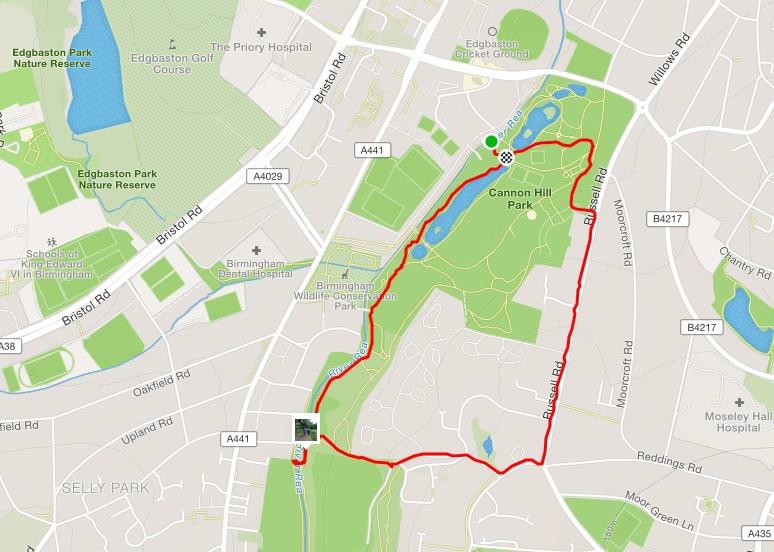


Walk in Cannon Hill Park


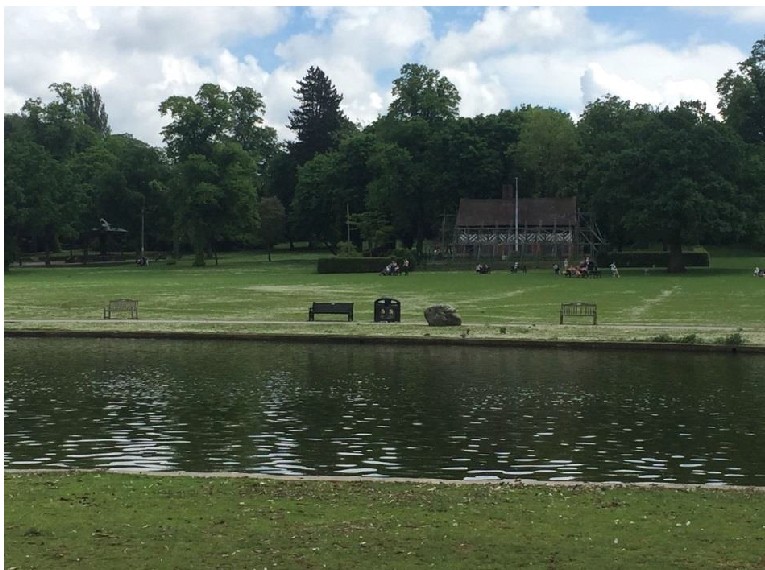

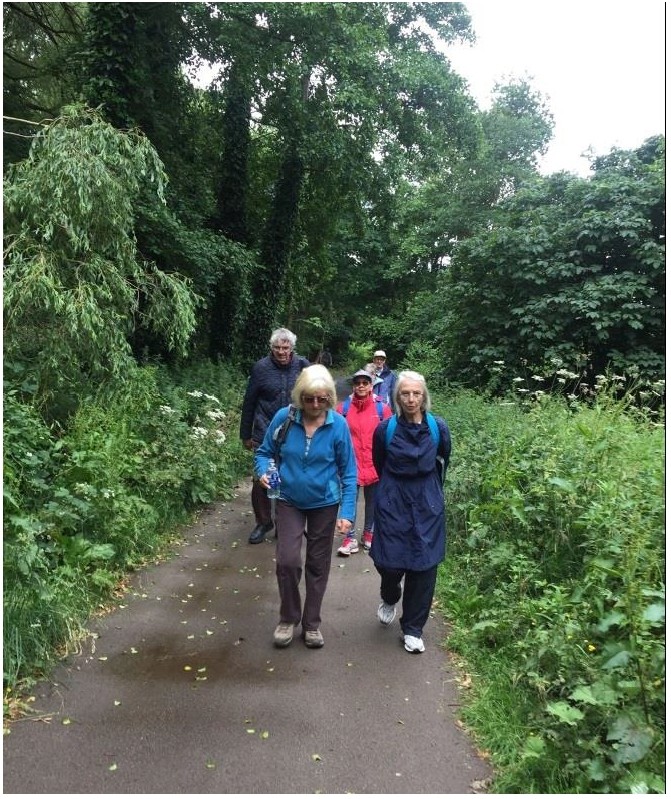


**Topic for discussion**


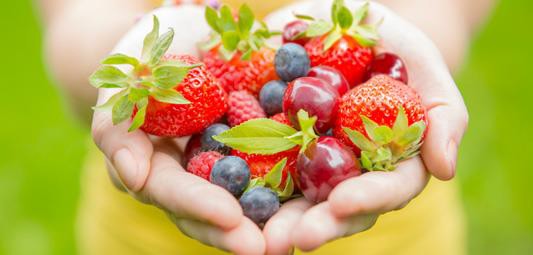

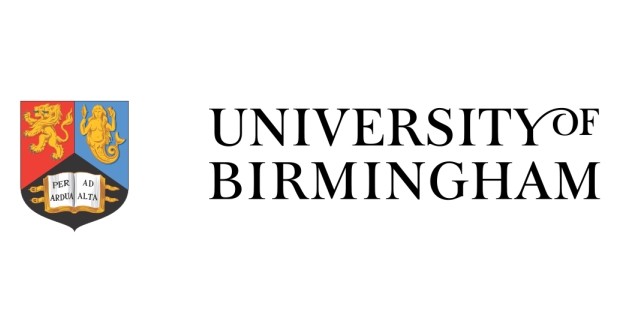


**Healthy eating: nutritional guidelines**

**for older adults**

**Week 3 –Warley Woods Park**


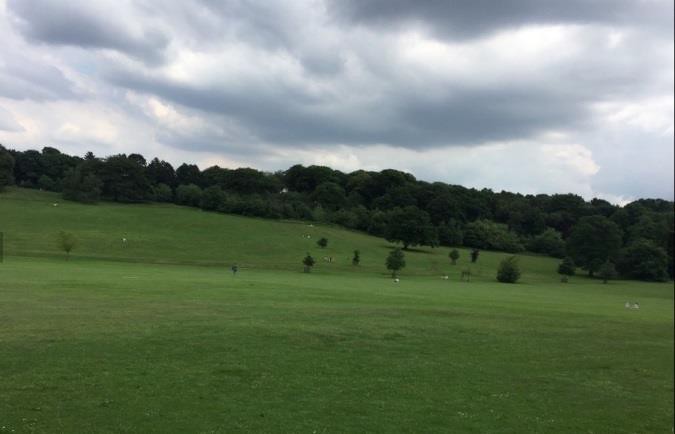


• Saturday 10.00am

• 30th of June

• Start and endpoint:

• Near Pavilion cafe The Pavilion, 101 Lightwoods Hill, Smethwick B67 5ED located off the Lightwoods Hill.

Getting there: From the University bus No48 to Hagley Road West and a short walk to the park. Car parking: The car park off Lightwoods Hill, near

The Pavilion.

* *Coffee stop at Pavilion cafe.* The [café](http://www.warleywoods.org.uk/?page_id=254) is open 9am – 3pm weekdays

**Warley Woods Park**

The Pavilion, 101 Lightwoods Hill, Smethwick B67 5ED.


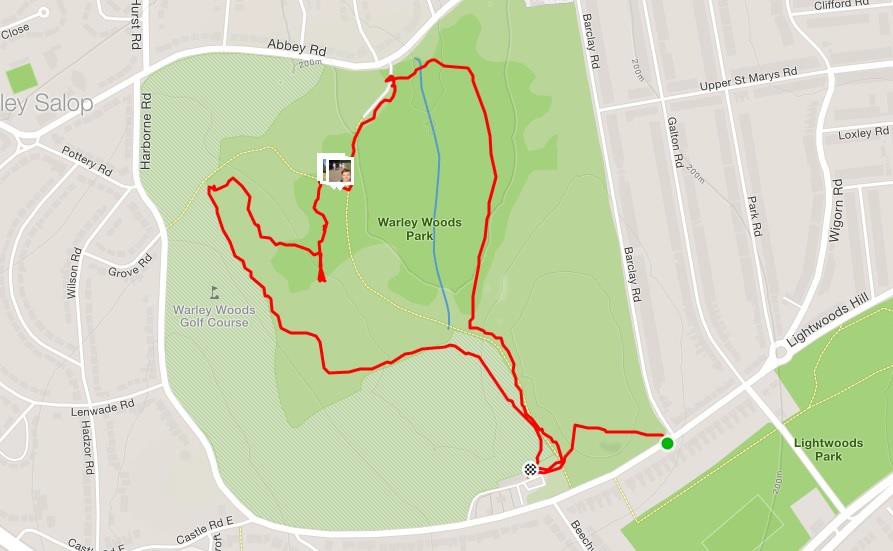


Walk at Warley Woods Park


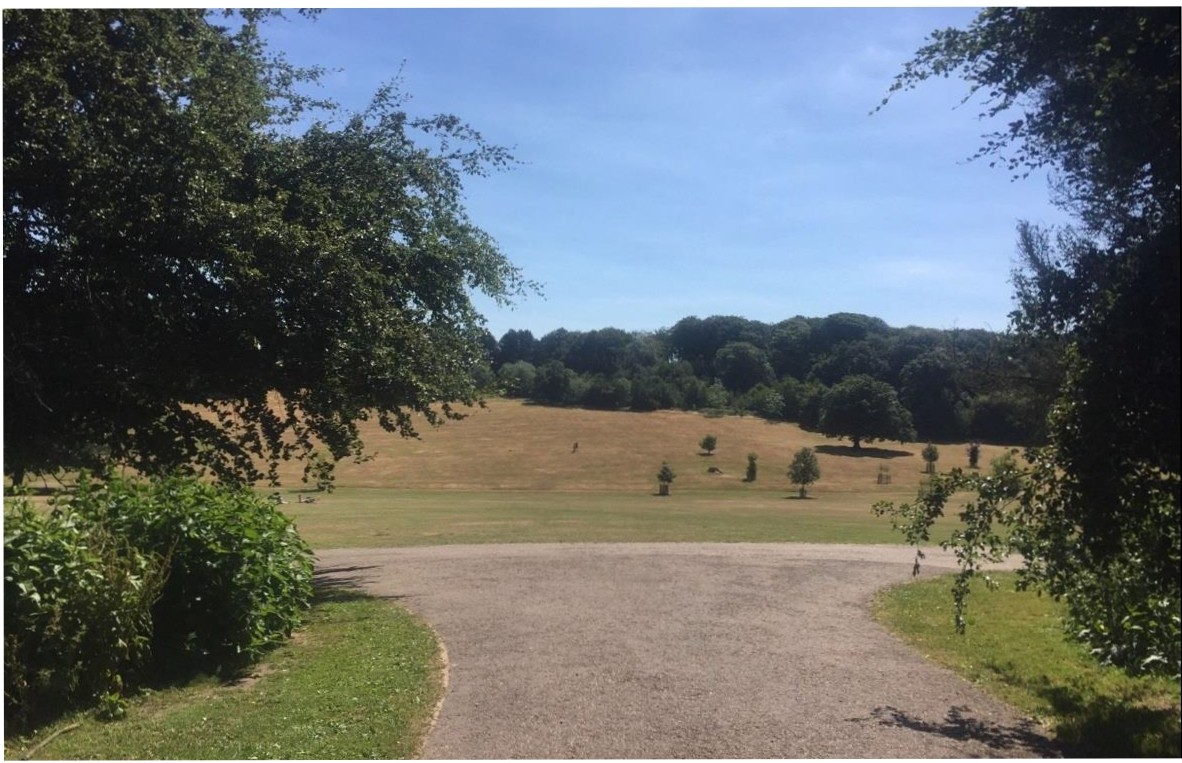


**Topic for discussion**


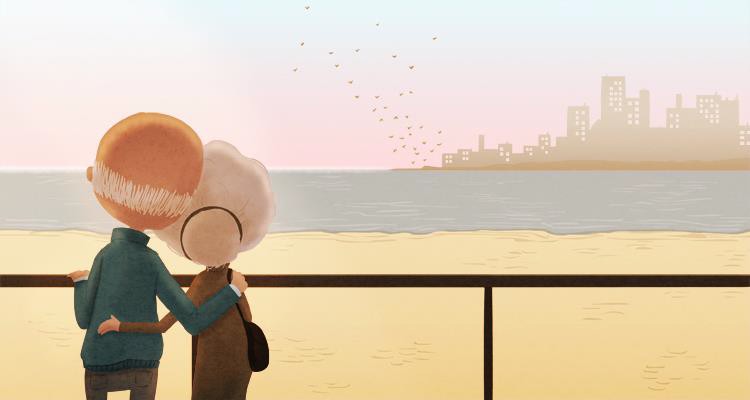

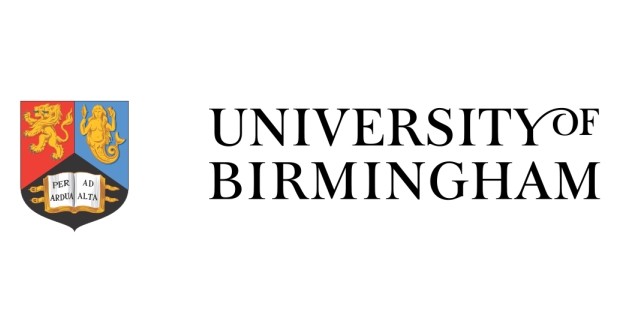


**Loneliness**

**Richmond Park & Isabella Plantation**

**(London) – Week 3 (optional)***

• Sunday 12.00am


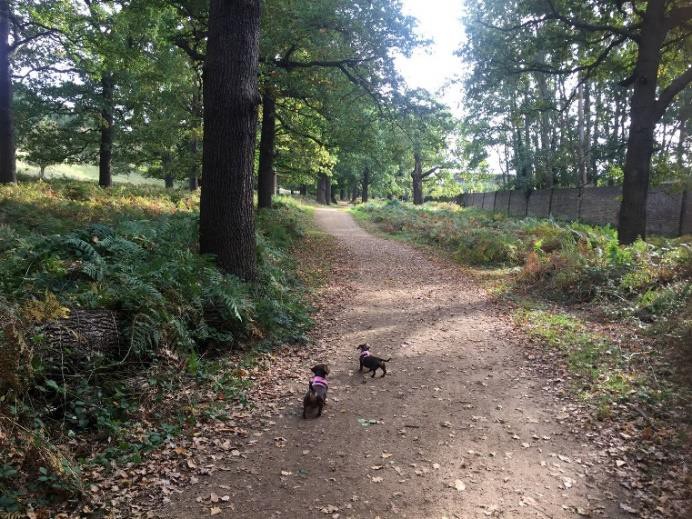


• 1st of July,

• Start and endpoint:

Kingston Gate

(Richmond park)

Getting there: Trains available from Vauxhall or Clapham Junction to Norbiton station (from there it is 10 minutes walk away to Kingston Gate

Parking is available at Kingston Gate (Richmond

park). Sat nav KT2 5JN

* *Coffee stop at Pavillion caffe.* The [café](http://www.warleywoods.org.uk/?page_id=254) is open 9am – 3pm weekdays

**Richmond Park & Isabella Plantations**

WALK 27 May 2018

| 2.8mi | **1:13:11** | 0 |
| --- | --- | --- |
| Distance | Moving Time | Calories |


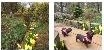


**Ham Common**

TerrainMap •

KINGSII.!JI


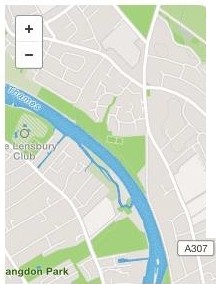

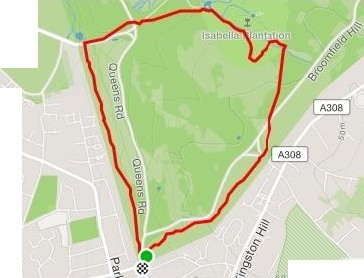


.. VAl E

--- • I

r. **Coombe HJII Golf Ctu6**

@Mapbox@ OpenStreetMap Improve this map


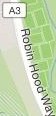


---

**Kingston Gate (Richmond park)**

**HAM** *Ric*.*hmond Park*


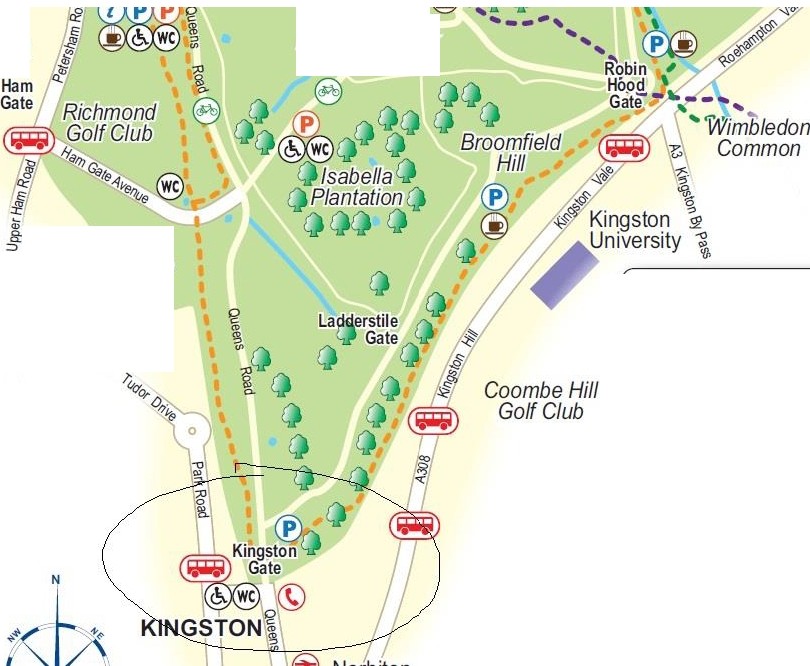


*Ham*

*Common*

**KEY TO MAP SYMBOLS**

••• ................Tamsin Trail

(Walking &cyding)

••• ....Beverley Brook Walk

- - - ...............Thames Path

--- .................CapitalRing

.............Railway station

**ClJ•LO...........**............Bus stop

®...........................Parking

®............Disabled Parking

@...................Public toilets

.........Disabled toilets

@................Refreshments

**Lickey Hills Country park– Week 4**

• Thursday 10.30-11.30am


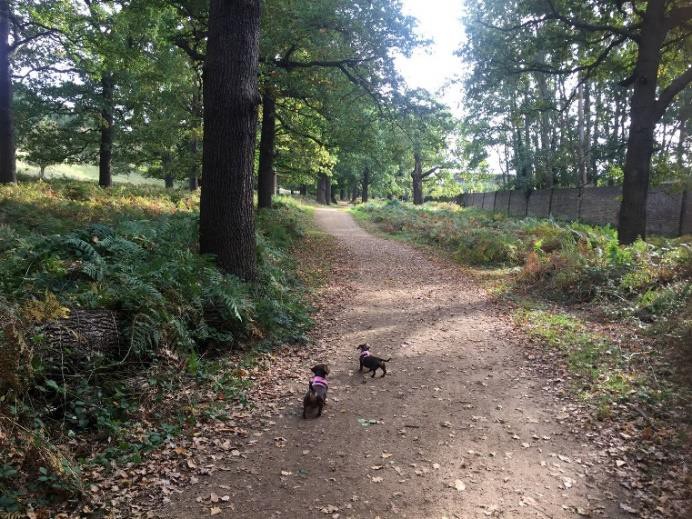


• 5th of July,

• Start and endpoint:

Visitor centre,

Warren Lane, Lickey, B45 8ER

Getting there: Train from University to Kings Norton, then bus 47 from Kings Norton to Rednal Island. Walk 5 minutes to the destination.

**Lickey Hills Country park**

*y*


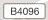


Bilbe"rr'y Hill


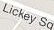


'


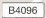


*)*


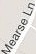


cotton FlacRett


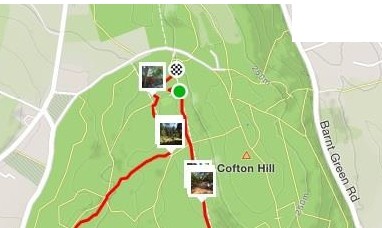

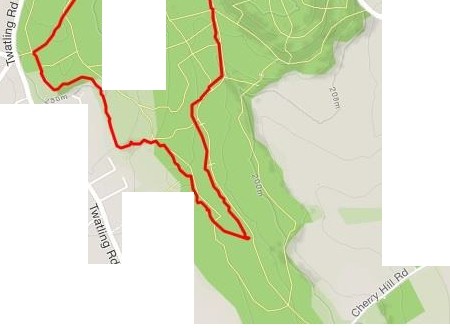

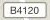

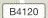

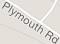

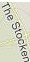

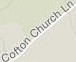

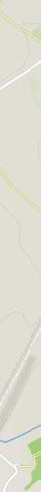


B45 8ER


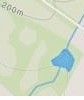


Walk at Lickey Hills Country Park


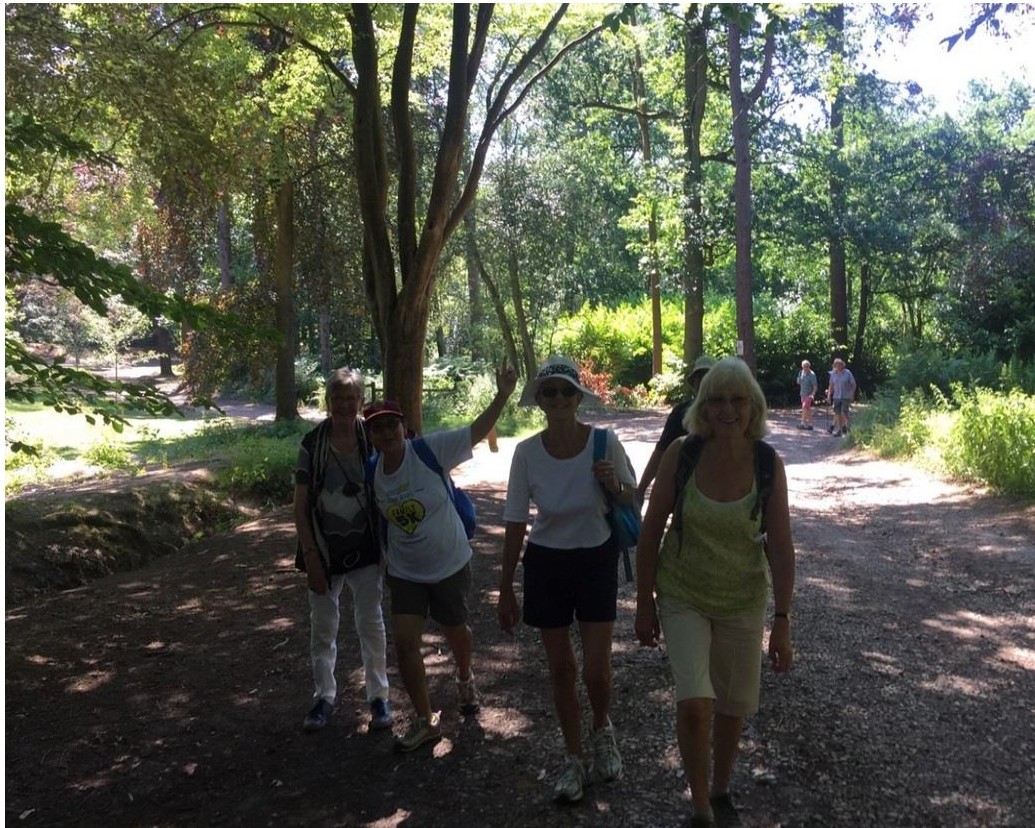


Walk at Lickey Hills Country Park


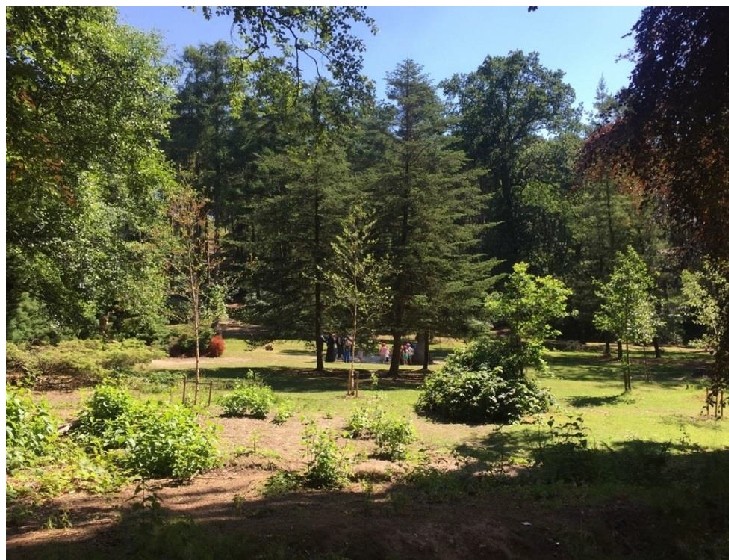

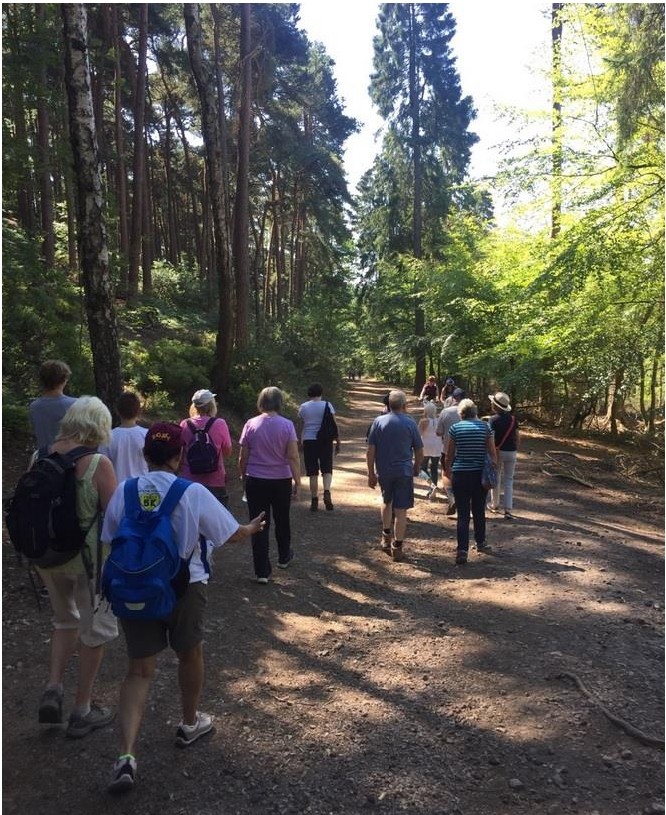


**Week 4 - Topic for discussion**


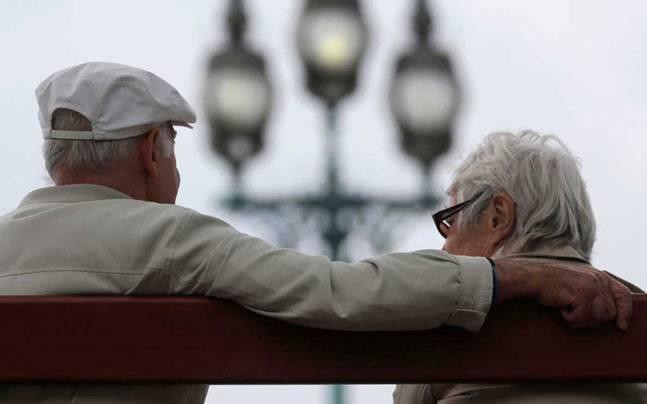

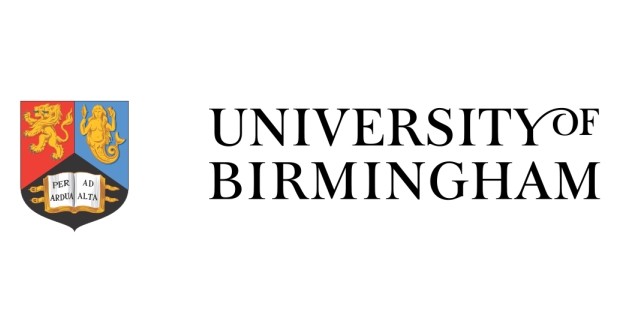


**Social support**

**Focus group interview**

**Wednesday 11th of July**

**11.30-12.30**

***1. Mid-point focus group interview (Approximate duration 1 hour)***

• To gather participants’ experience and attitudes of taking part in the

PAIL study

***Where:***

***Room G05 (ground floor left block, School of Sport, Exercise and Rehabilitation Sciences, University of Birmingham)***


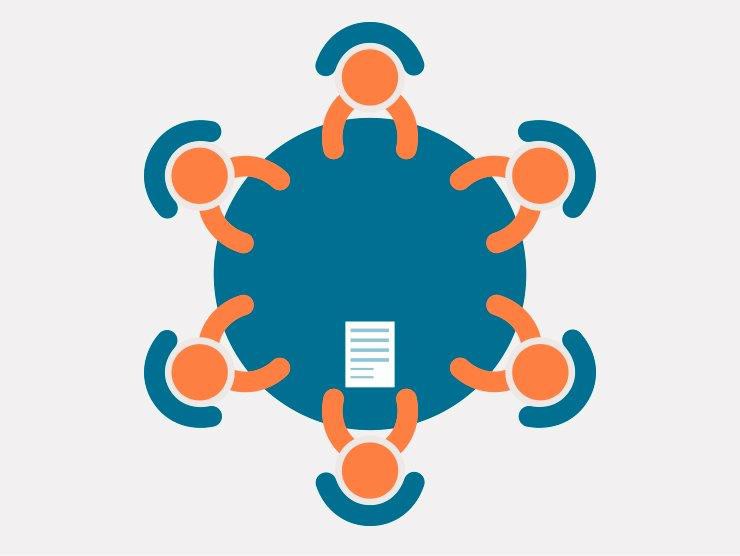


**Week 5 - Vale & Winterbourne House**

**and Gardens**


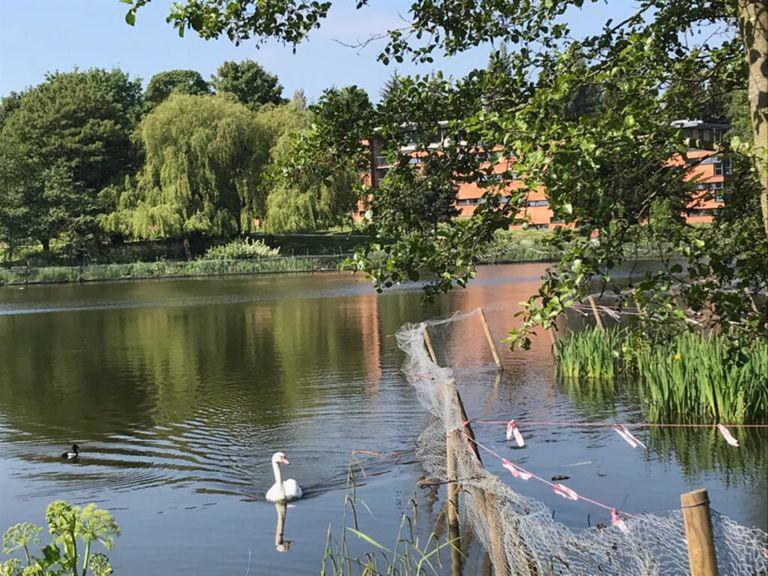


• Saturday 10.00am

• 14th of July,

• Start and endpoint: University of Birmingham train station

• Walk along the canal up to the Vale

• Getting there: All bus and train routes to the

University train station

• Parking available free of charge near University

train station

* *Coffee stop at Winterbourne Gardens (free entry)*

**Vale & Winterbourne House**

**and Gardens**


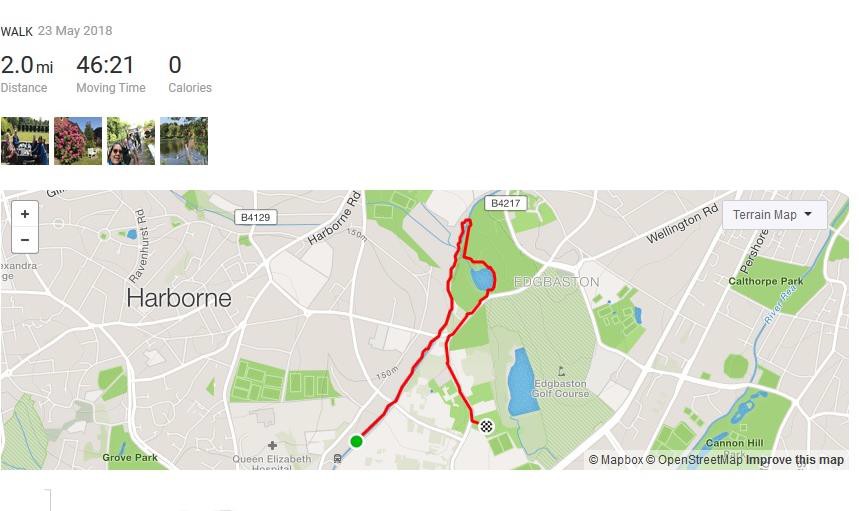


At Winterborne House and Gardens


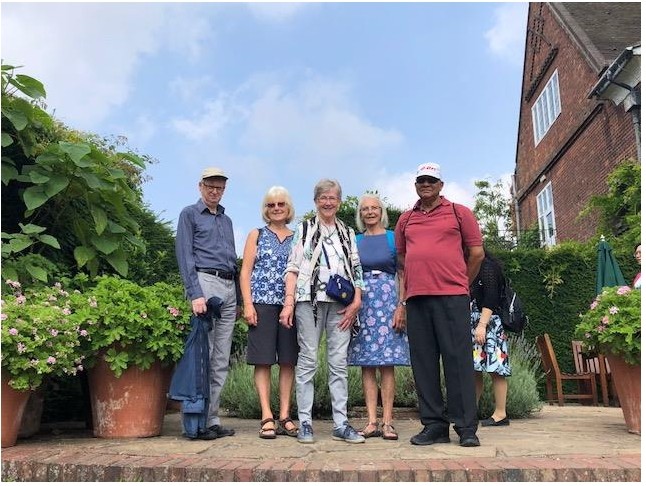

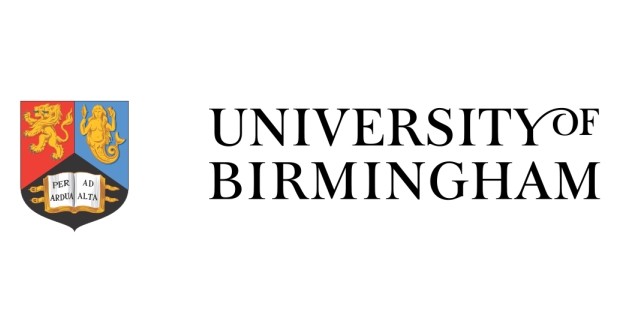

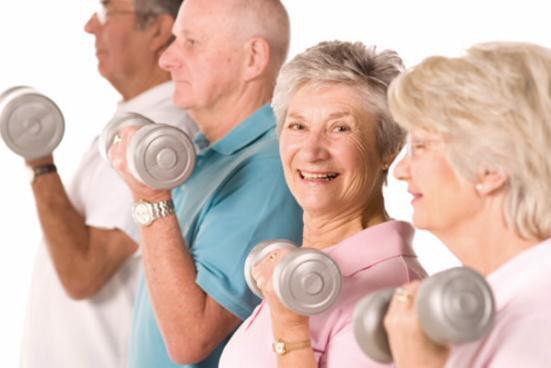


**Week 5 - Topic for discussion**

**Physical activity for older adults**

**Week 6 – Woodgate Valley Country**

**Park**

• Saturday 10.00am

• 21st of July,

• Start and endpoint:

Woodgate Valley Visitor’s centre, B32 3DS

• Getting there: All bus and train routes to the

University train station

• Parking available at the car park B32 3DS

| **Topic** | **for discussion** |  |
| --- | --- | --- |
| **Local** | **events and exercise** | **activities** |

https://marketplace.mycarein birmingham.org.uk/Activity/C ategory/13


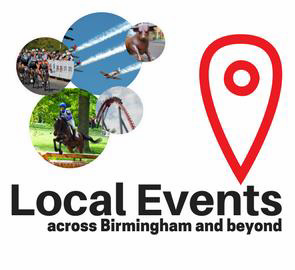


Woodgate Valley Country Park

ourD !i

AcadeiiiW


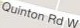


Simmons *Dr*

Quinton *Rd* w


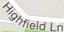

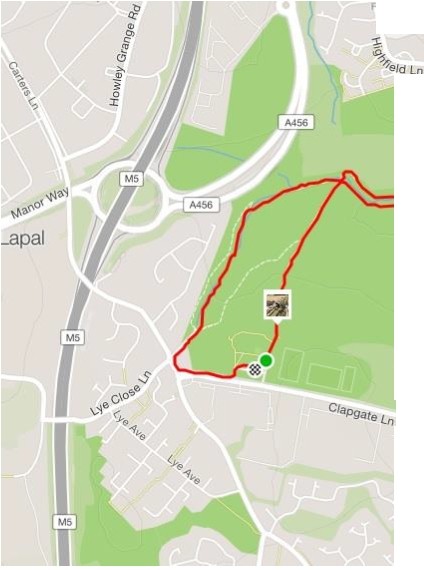

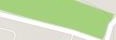

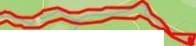


Woodgate Valley

Country Park

cpnmerll8ldRd


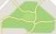

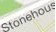


*Clan-.*


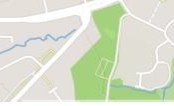

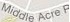


*-,ate Ln*


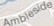


**Walk at Woodgate Valley Country**

**Park**


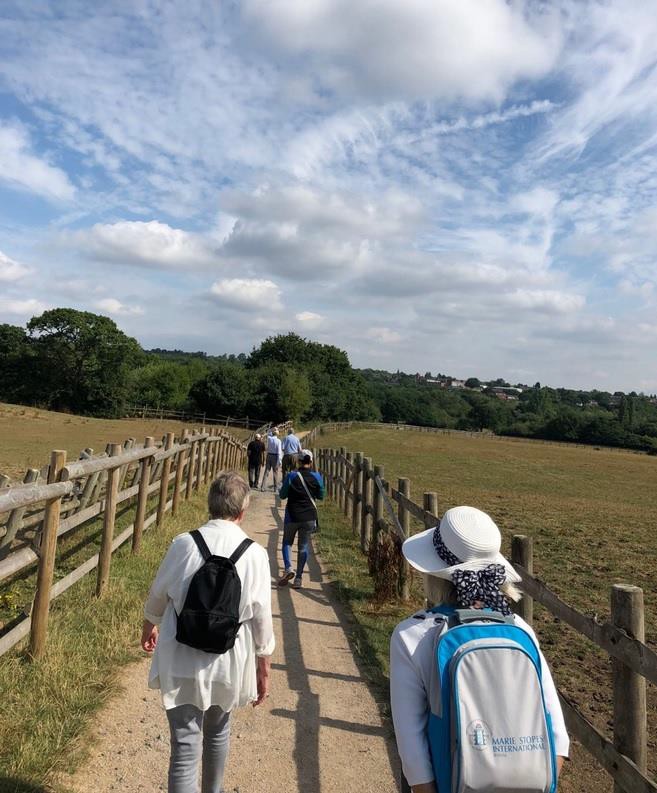

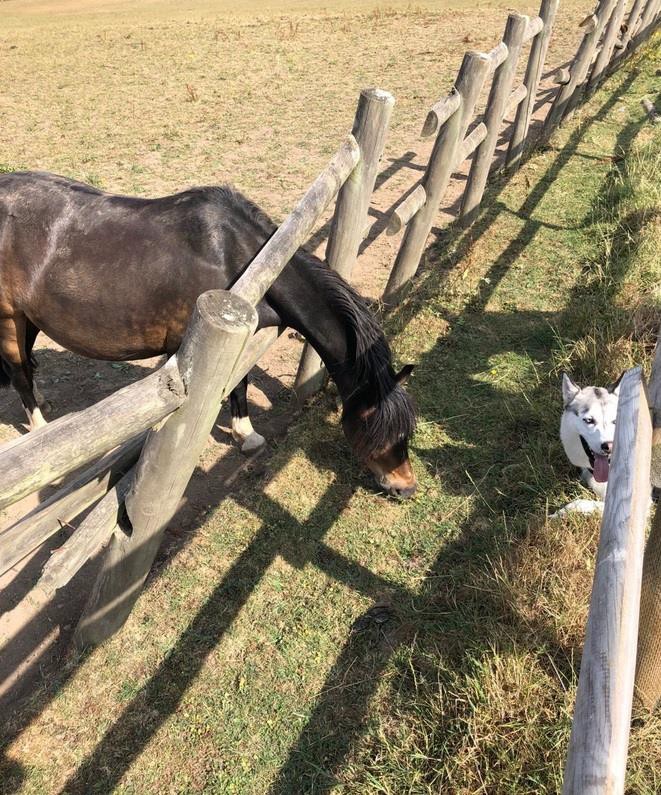


**Walk at Woodgate Valley Country**

**Park**


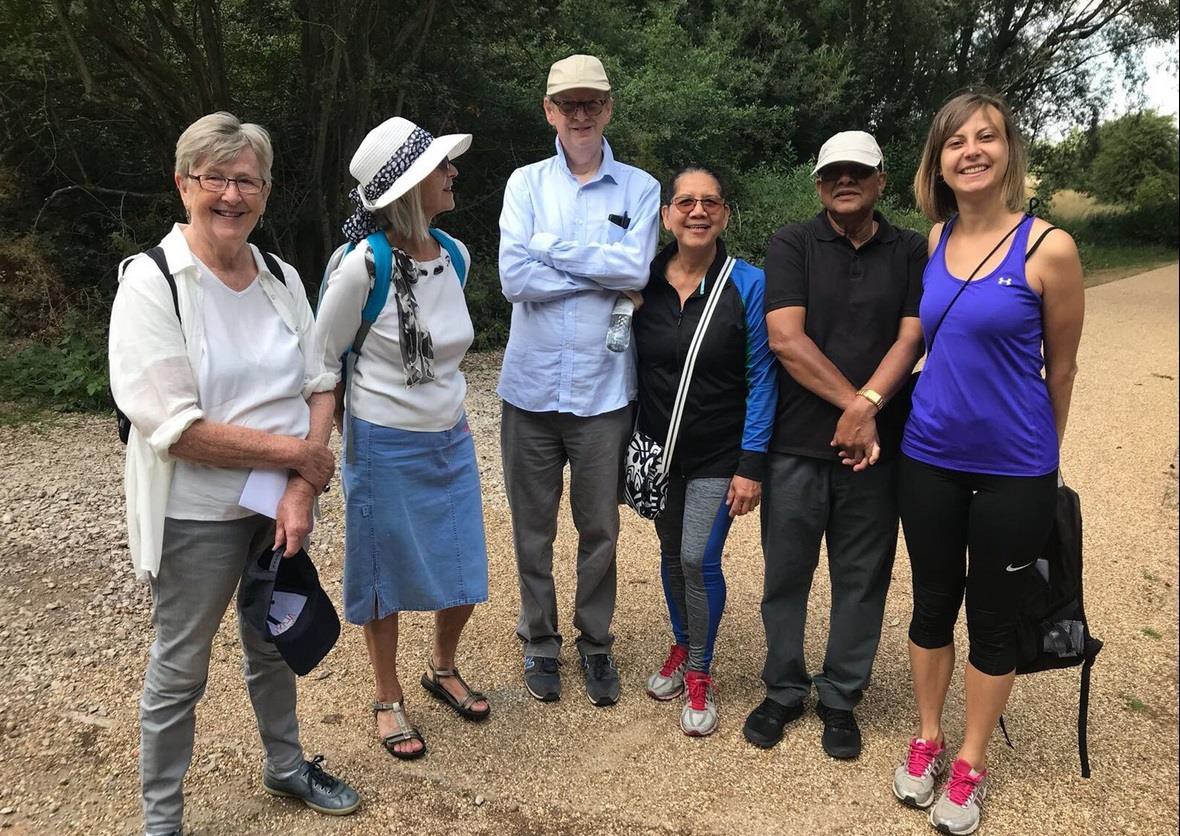


**Week 7 – Vale & Winterbourne House**

**and Gardens**


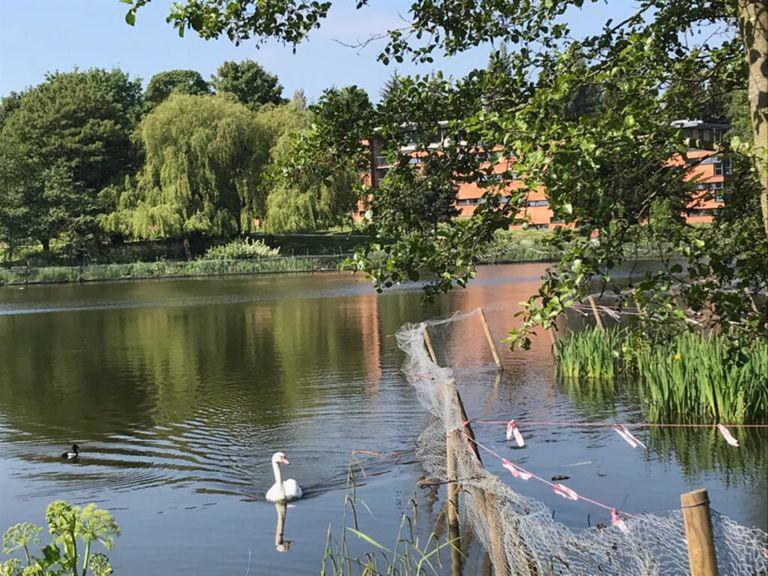


• Wednesday 11.00am

• 25th of July,

• Start and endpoint:

University of Birmingham

train station

• Walk along the canal up to the Vale

• Getting there: All bus and train routes to the University train station

• Parking available free of charge near University train

station

* *Coffee stop at Winterbourne Gardens (free entry)*

**Vale & Winterbourne House**

**and Gardens**


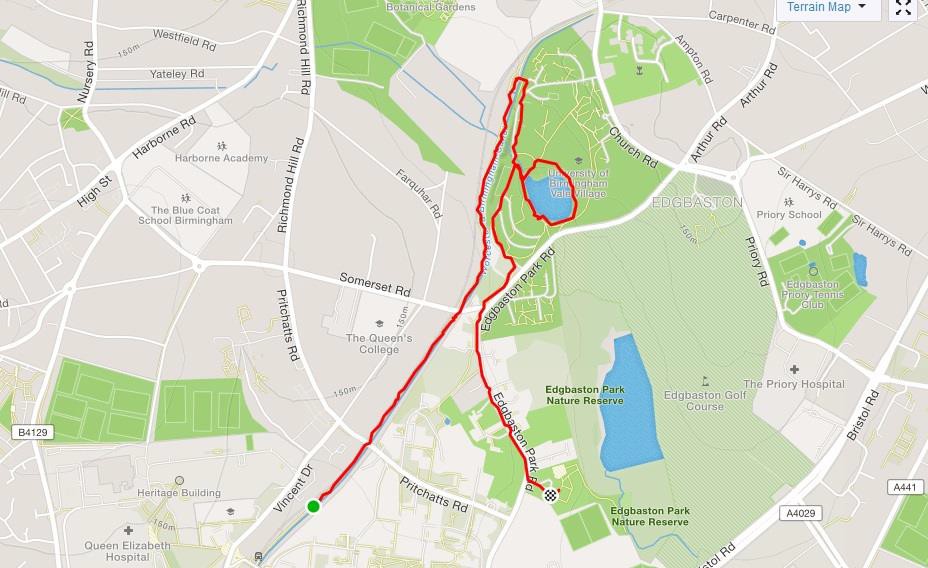


**Topic for discussion**


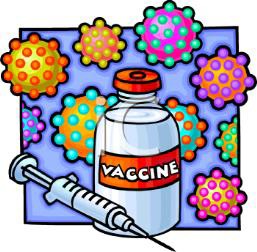


**The importance of**

**the ‘flu vaccination**


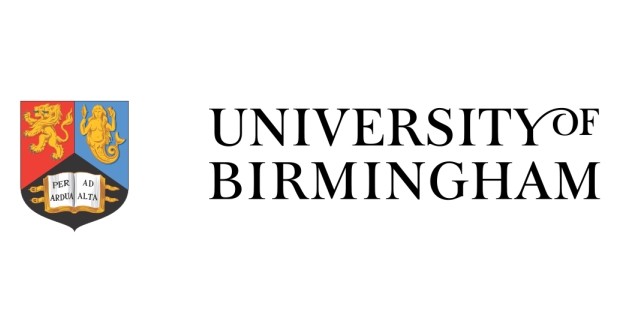

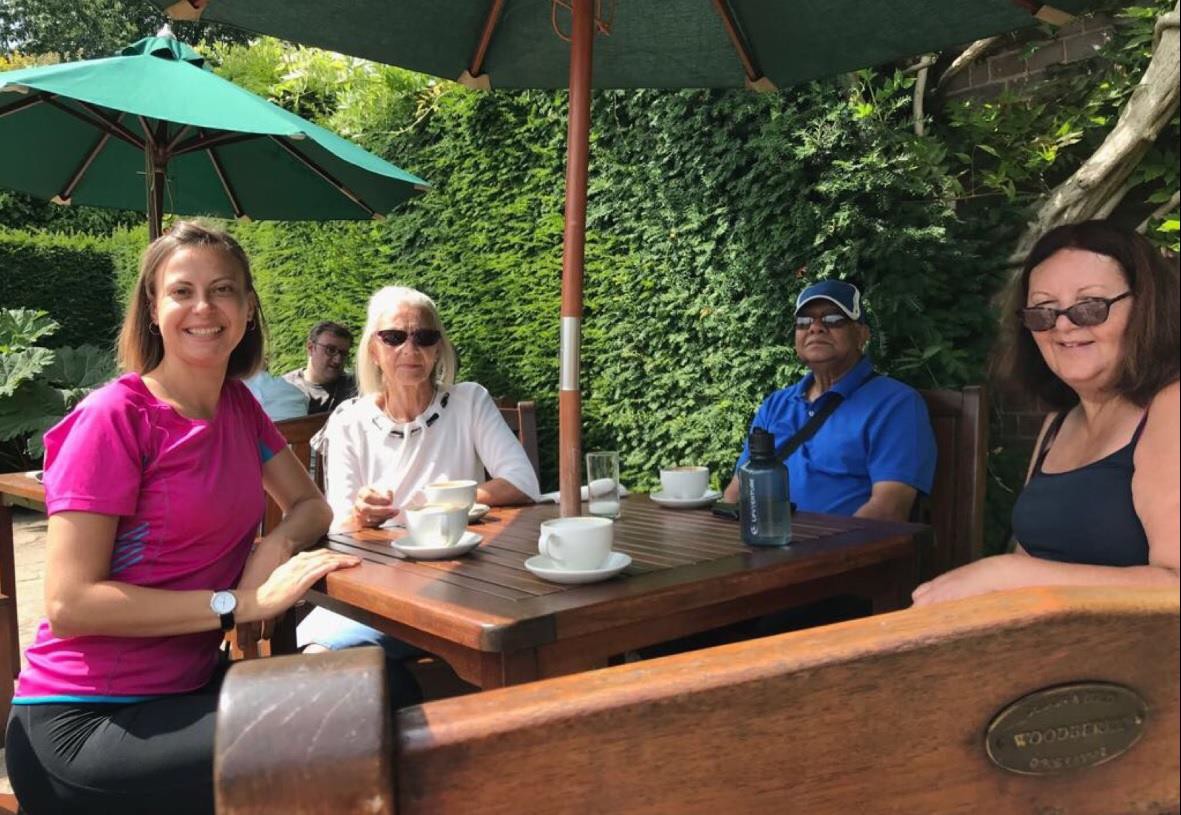


**Week 8 – Walk and Pizza at Plough**

**(Harborne)**


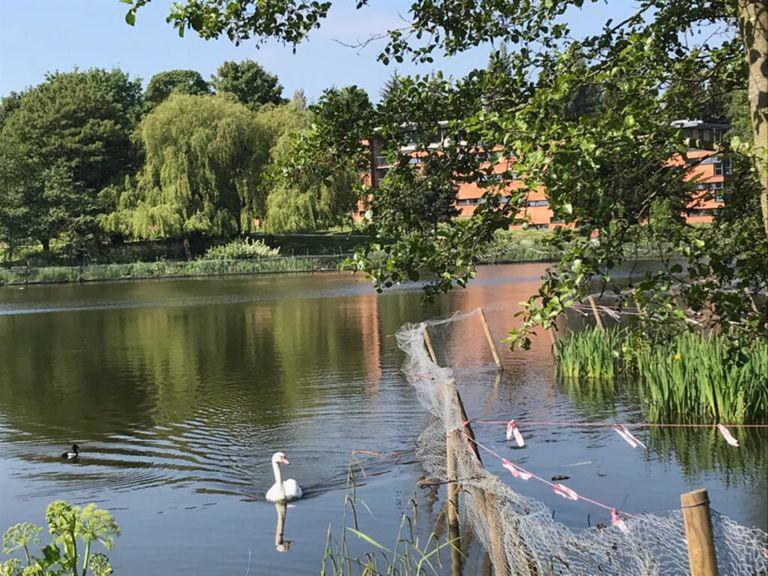


• Wednesday 11.00am

• 1st of August,

• Start point:

University of Birmingham train station

• Walk to Metchley Park & Groove Park and up to

Harborne

• Pizza at Plough bar (Harborne High street) 21 High St, Harborne, Birmingham. **B17 9NT**

**Topic for discussion**


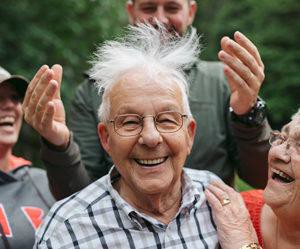

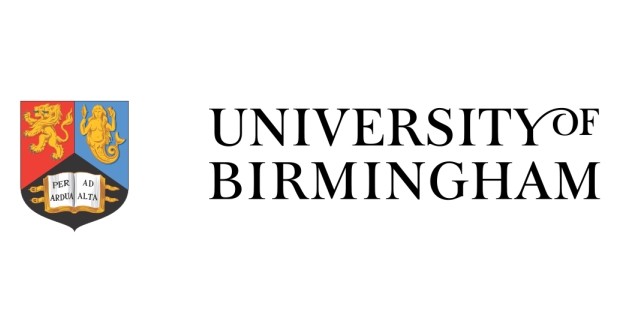


**Mental health and Wellbeing**


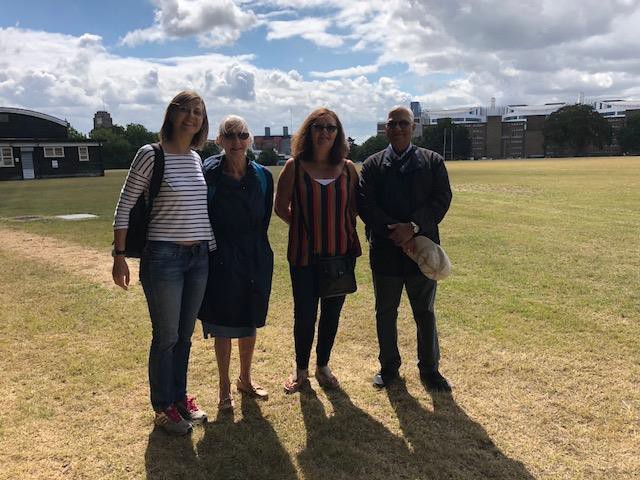

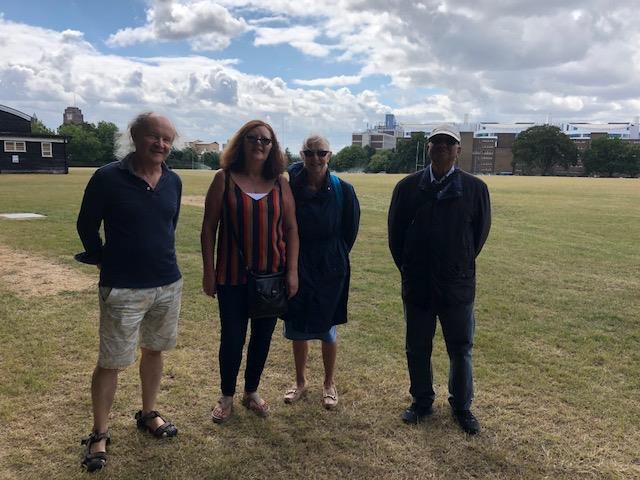

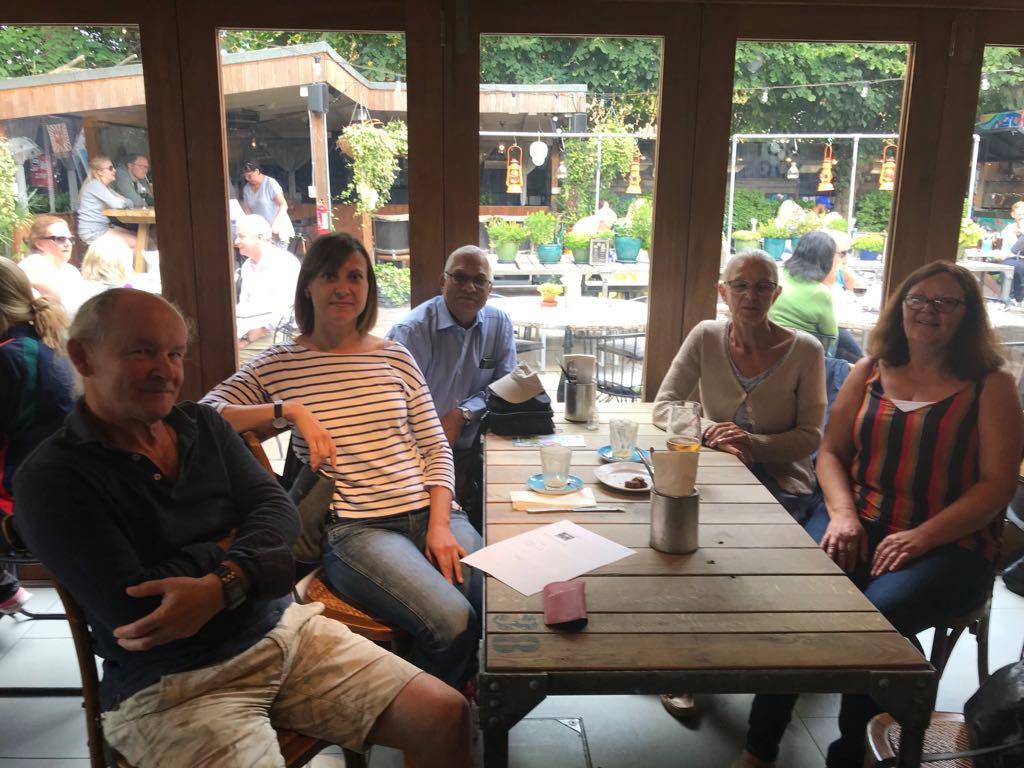


**Week 9 - Cannon Hill park**


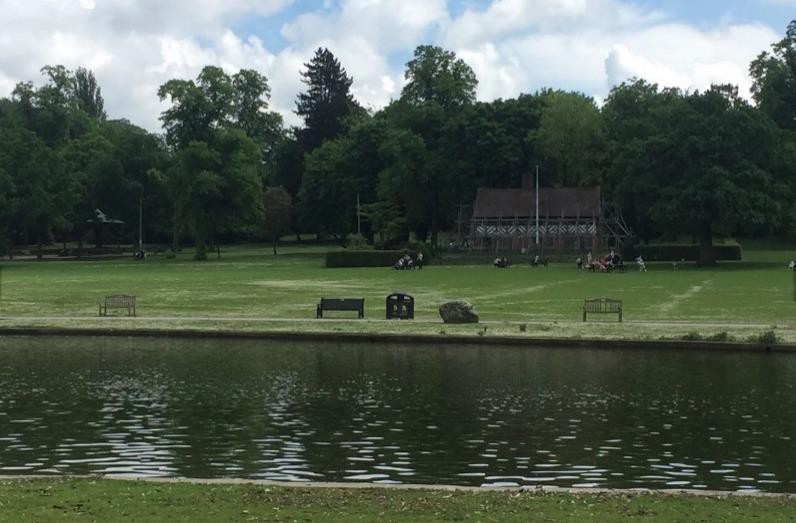


• Wednesday 12.00am

• 9th of August,

• Start and endpoint:

MAC, the Cannon Hill Park

• Getting there: From Selly Oak – bus No63 (Bristol

Road/Dawlish road) to Bristol Road/Priory Road.

• Bus X61, 144 from Bristol Road.

• Parking: There are two car parks, one by the MAC off Edgbaston Road £2 up to 4 hours, and a smaller car park off the Russell Road.

• *Coffee stop the Mac café.*

**Cannon Hill park walk**

**Mac,** Cannon Hill Park Queen's Ride, Birmingham B12 9QH


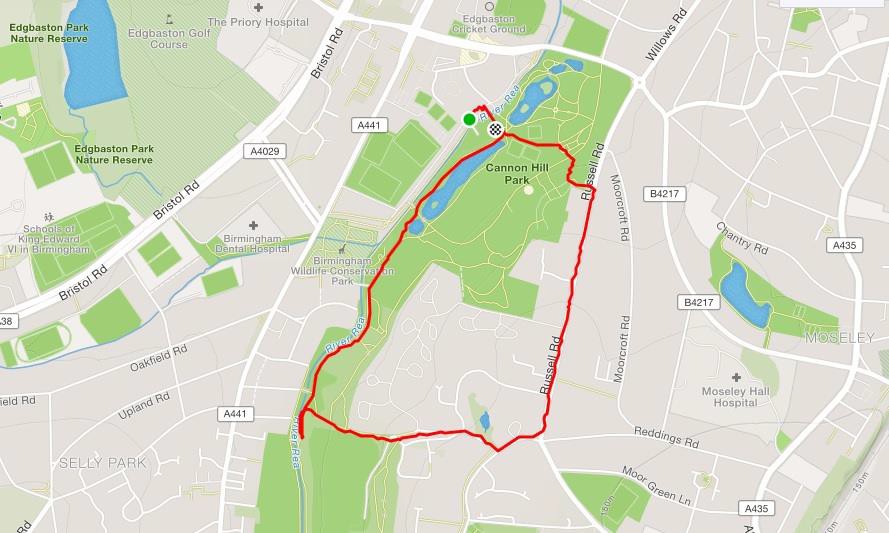


**Topic for discussion**


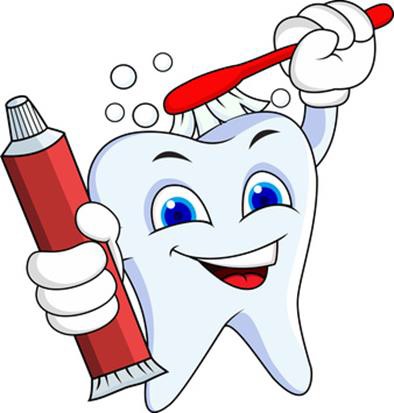


**Dental hygiene**


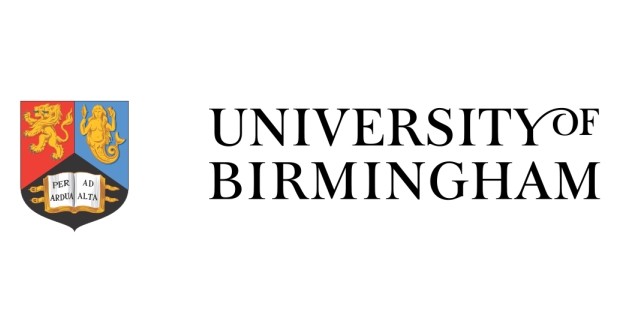


**Focus group interview**

**Tuesday 14th of August**

**13.15-14.15**

***1. End-point focus group interview (Approximate duration 1 hour)***

• To gather participants’ experience and attitudes of taking part in the

PAIL study

***Where:***

***Room G05 (ground floor left block, School of Sport, Exercise and Rehabilitation Sciences, University of Birmingham)***


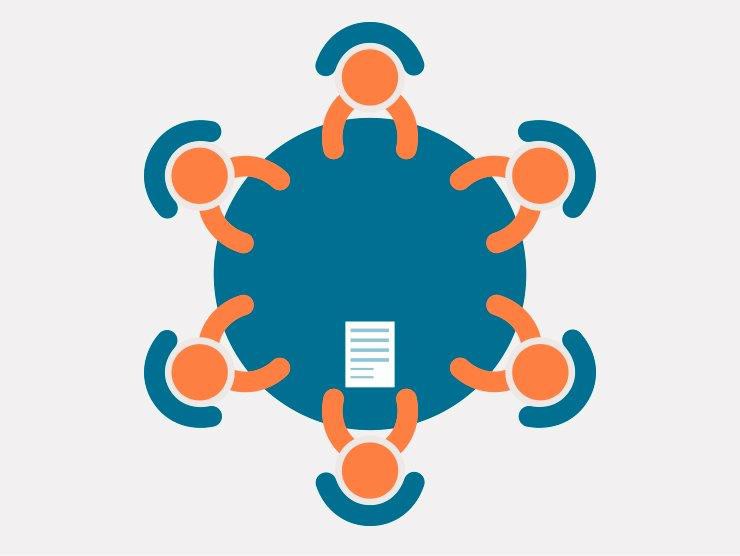


**Week 10 – Walk to the Vale**

• Saturday 10.00am


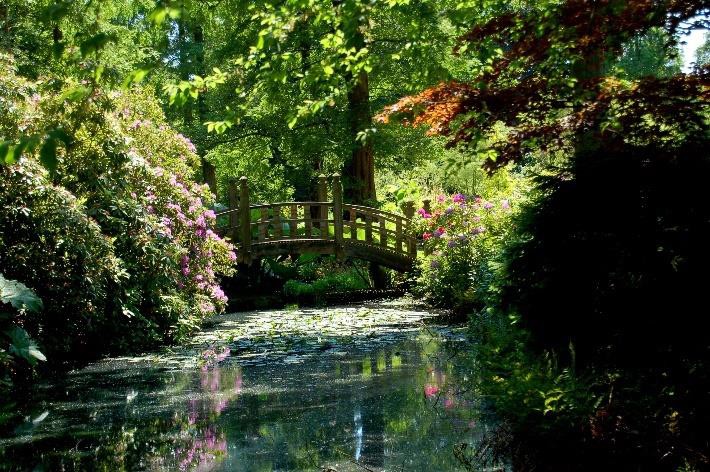


• 18th of August,

• Start and endpoint: University of Birmingham train station

• Walk from the University station along the canal

up to the Vale

• Getting there: All bus and train routes to the

University train station

• Parking available free of charge on campus

* *Coffee stop on the campus*


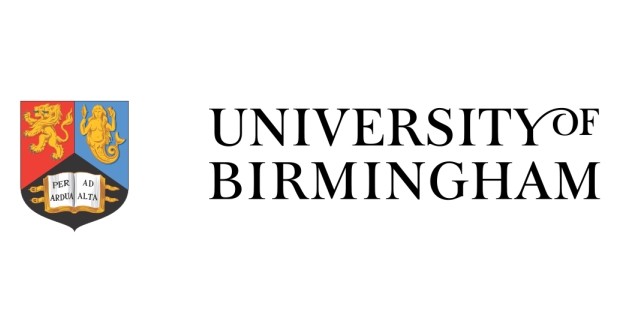

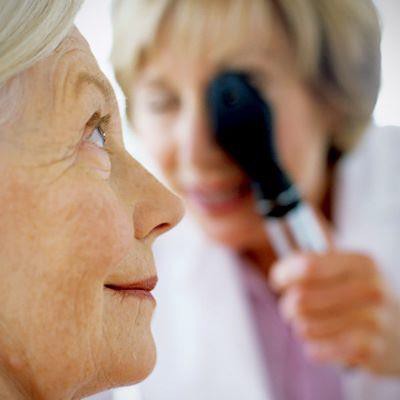


**Topic for discussion**

**Eye hygiene**

Walk along the canal


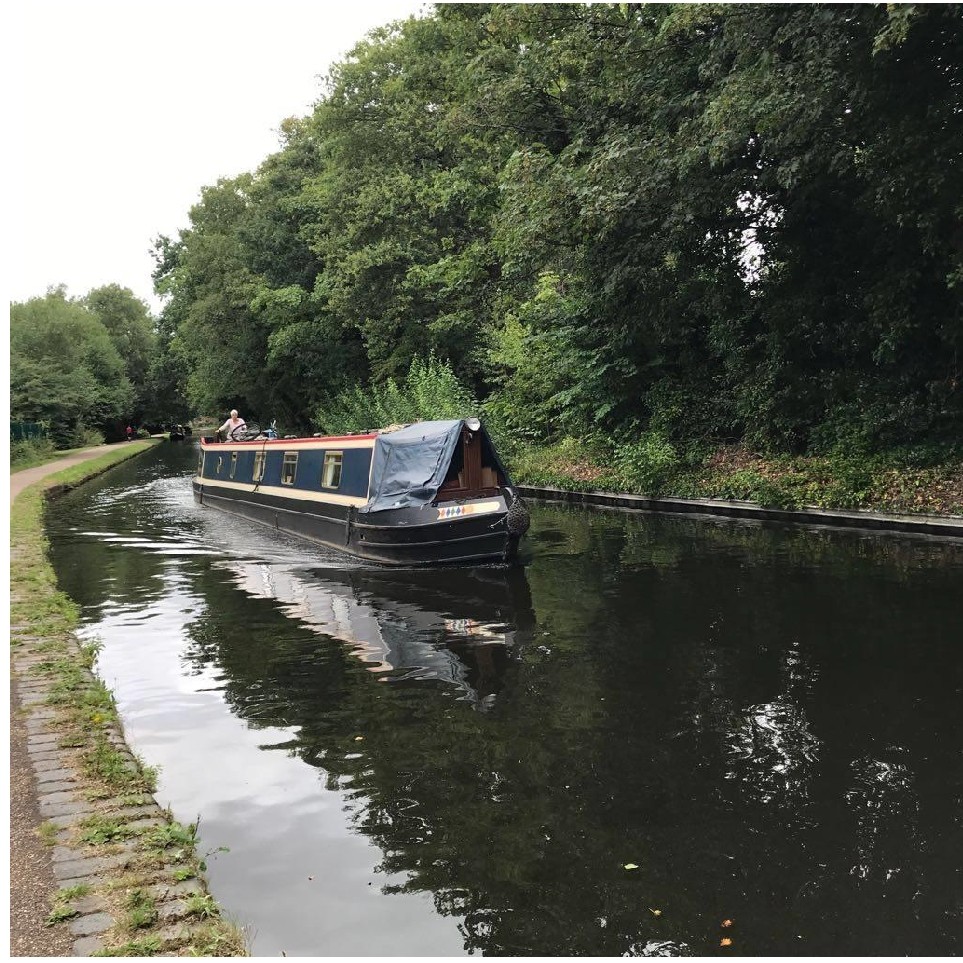

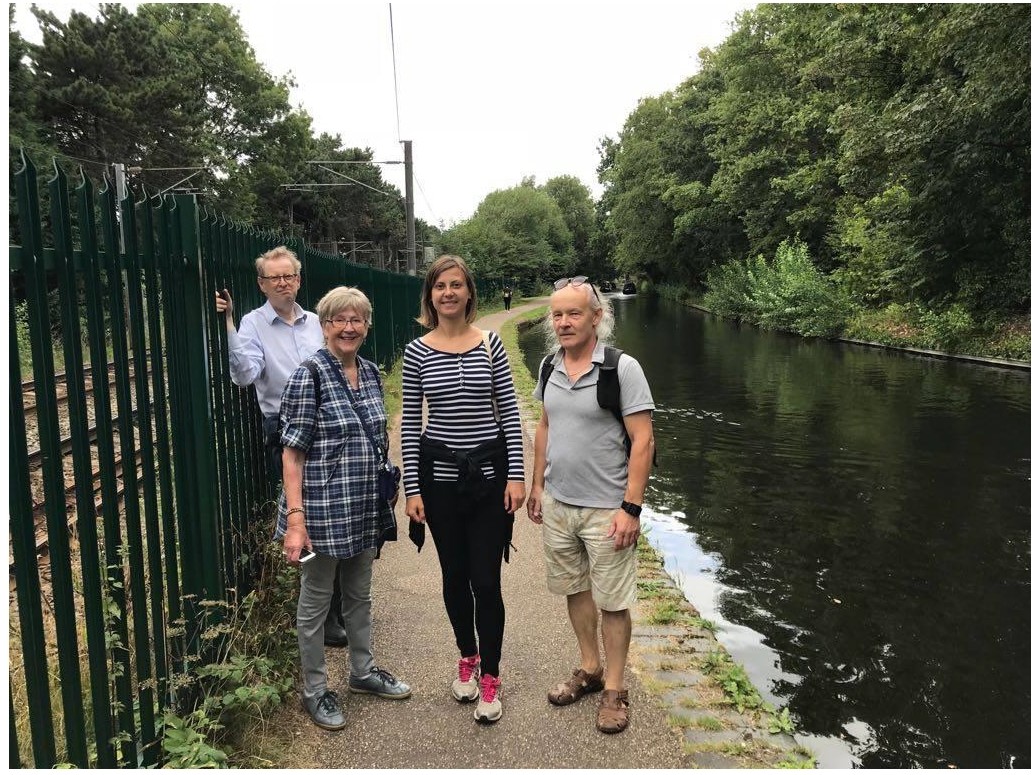


**Week 11 –Sandwell Valley Park**


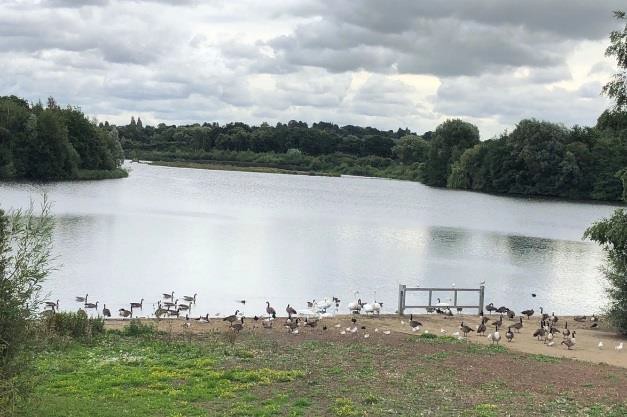


• Friday 11.30-12.30

• 24th of August

• Start and endpoint:

• Near RSPB Sandwell Valley B43 5AG, Panhouse

Avenue

Getting there: Bus No16 from Priory Queensway to Tanhouse Avenue, B43 5AG (£4 day saver or free with a senior bus pass)

Car parking: The car park at RSPB cost £2.

* *Coffee stop at RSPB caffe.*

Bird watching at RSPB Sandwell Valley


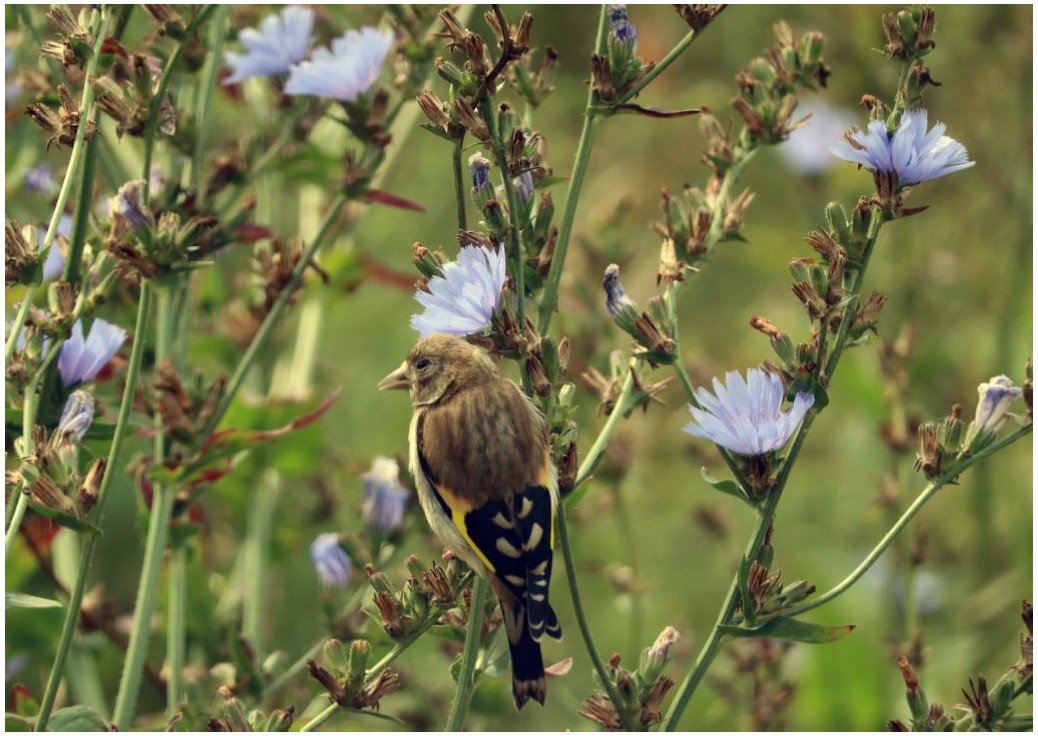


Photos by Patrick Anderson [patrick@vsnl.com](mailto:patrick@vsnl.com)

Bird watching at RSPB Sandwell Valley


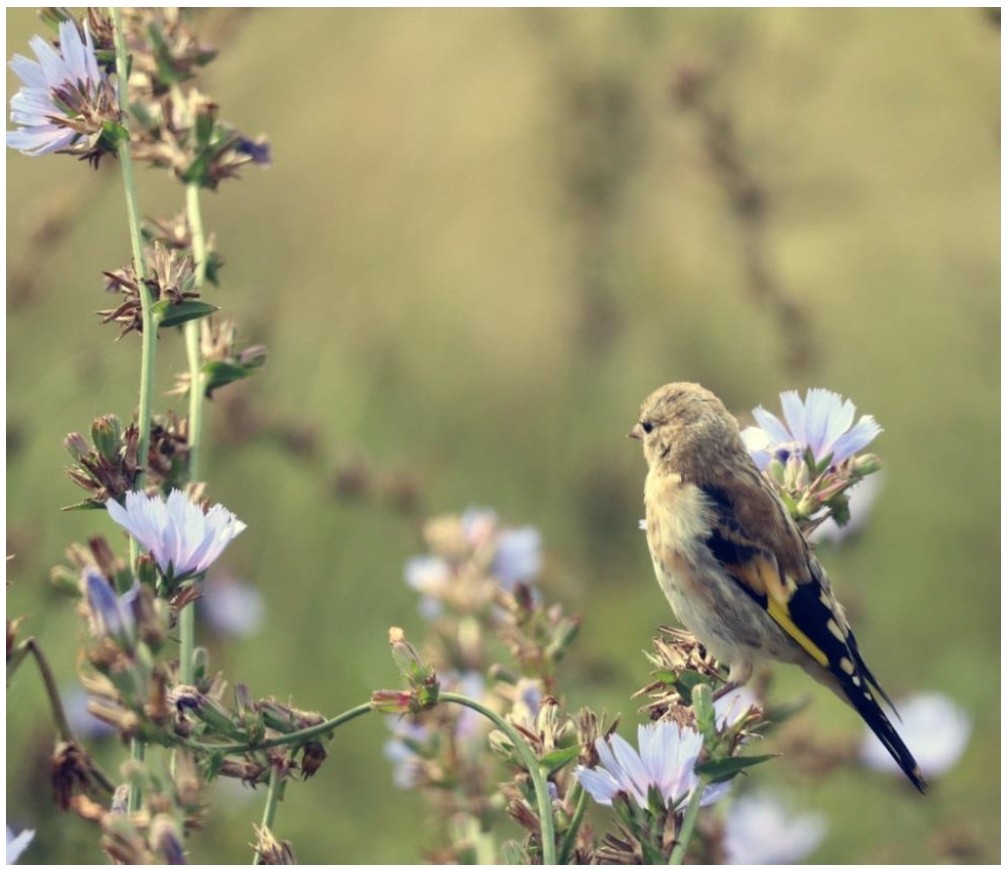

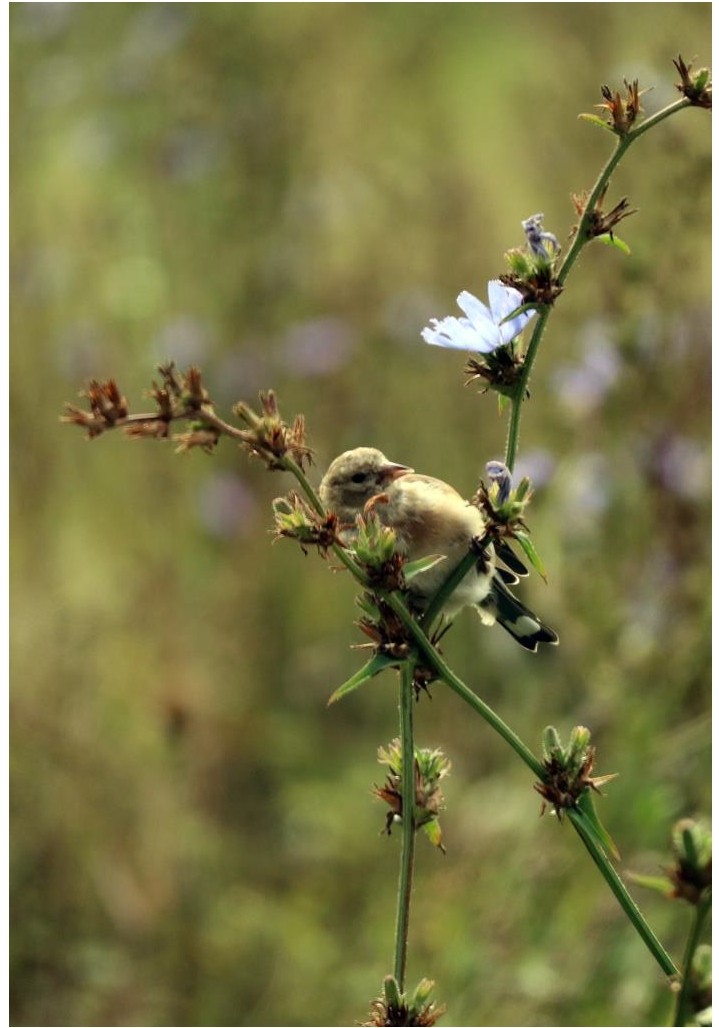


Photos by Patrick Anderson [patrick@vsnl.com](mailto:patrick@vsnl.com)

**Walk around Forge Mill Lake**

Sandwell Valley

Country Park

0

Ham$teacJ

Infants School

»- Hamstead Jumor School


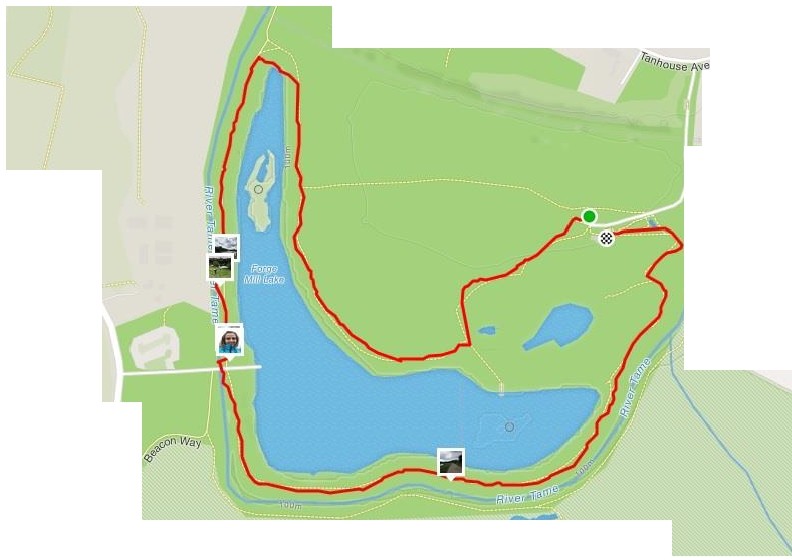

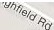

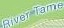

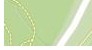


• Om

'*(*

"

c

*...J*

Q'"

Walk around Forge Mill Lake


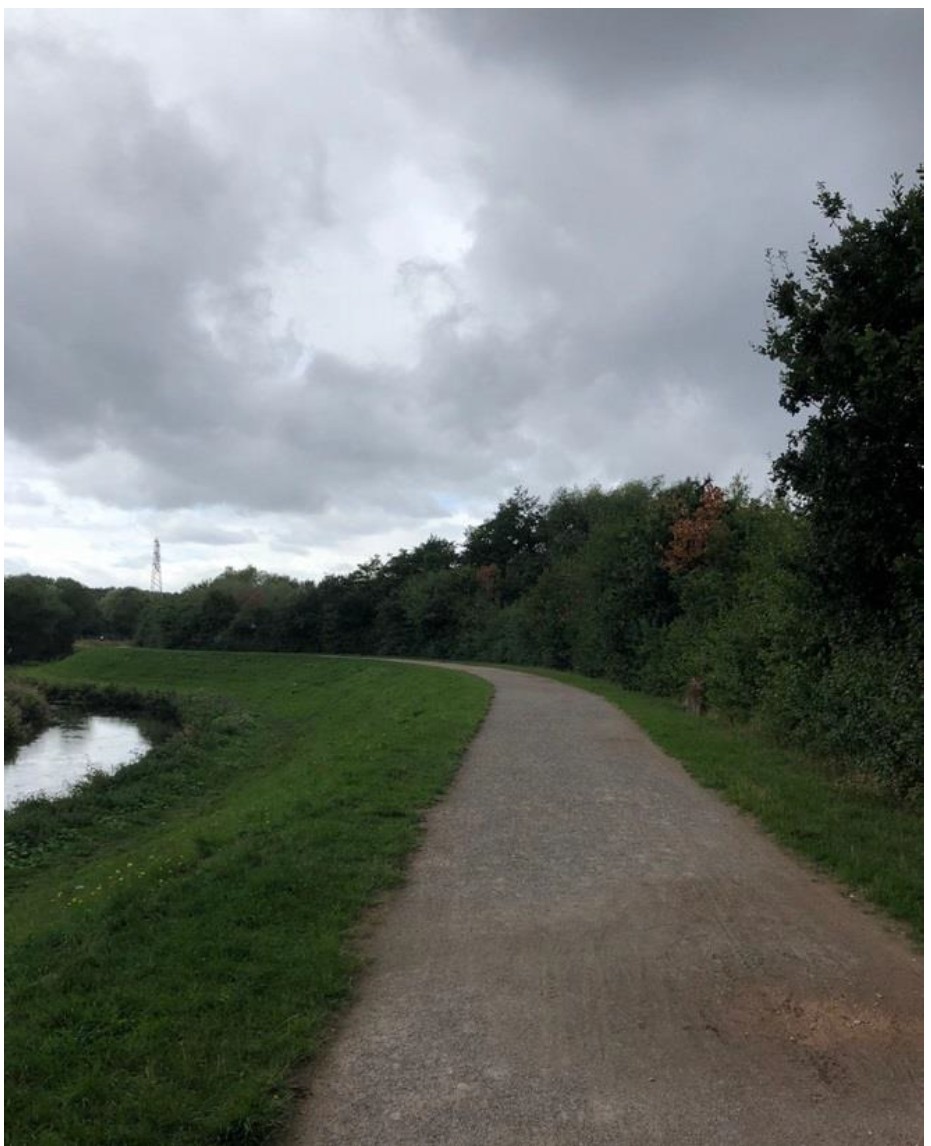

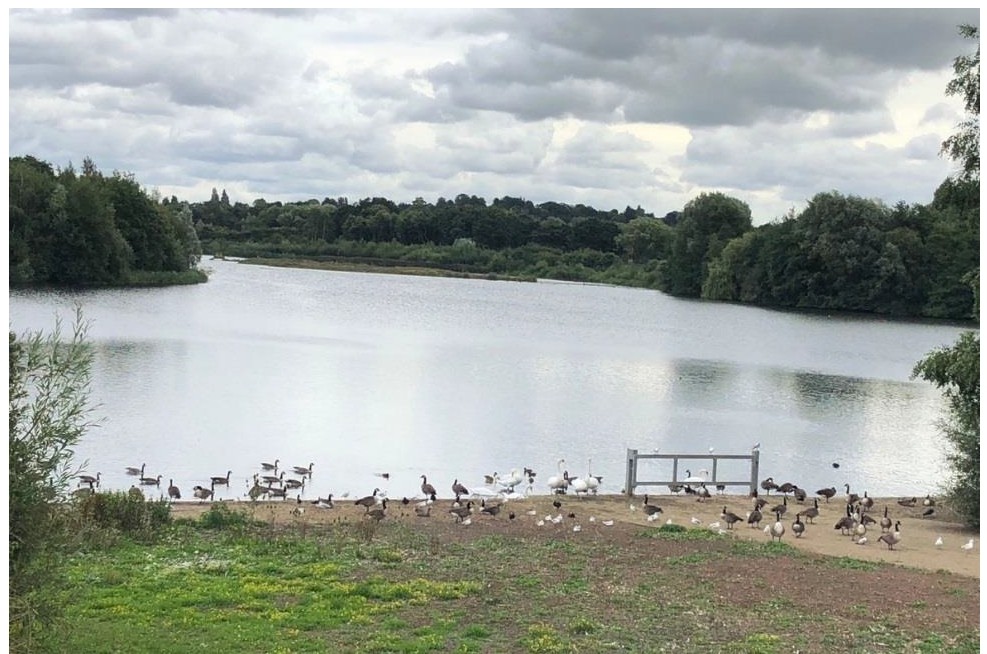


**Topic for discussion**

**Keep active**

**Week 12 - Vale & Winterbourne**

**House and Gardens**

• Wednesday 11.00am

• 29 of August

• Start and endpoint: University of Birmingham train station

• Walk along the canal up to the Vale

• Getting there: All bus and train routes to the

University train station

• Parking available free of charge near the

University train station

* *Coffee stop at Winterbourne Gardens (free entry)*

**Vale & Winterbourne House**

**and Gardens**

**Topic for discussion**

**Preventing the risk of falls**

**Thank you for being with us all this time!**

Anastasia Shvedko

**mob.** 07490392720

**e-mail:** [axs1235@bham.ac.uk](mailto:axs1235@bham.ac.uk)
