# Supplementary material for: Physical Activity Intervention for Loneliness (PAIL) in community-dwelling older adults: a randomised feasibility study
Source: Pilot Feasibility Stud. 2020 May 23;6:73. doi: 10.1186/s40814-020-00587-0 (PMC7245022; doi:10.1186/s40814-020-00587-0)
Supplement: Supplementary file 4 — Additional file 4. Main themes and emerged sub-themes from the mid-point focus group interviews [file 40814_2020_587_MOESM4_ESM.docx]

**Additional file 4** Main themes and emerged sub-themes from the mid-point focus group interviews

| **Themes and sub-themes** | **Quotes** |
| --- | --- |
| ***Theme 1. Study design and recruitment*** |  |
| Reasons for participation | Reason for participating in the intervention were to ‘keep going’ and make an effort to get out and about to be active and healthy: *“(.) I think you know there are all these motivations and keeping going, you have got to keep going. You have got to keep going. And it is an effort, and as you get older it is more of an effort, that you have got to do it if you want to be well”* (Alison, 75, female). |
| Participants | Participants expressed that, in particular, a wide range of ages and cultural diversity makes a significant difference in participants’ experiences and can enrich the group both socially and culturally: *“You will learn more. You know, from different people. I mean, I lived all over the world, but you learn more from different people and different cultures and different attitudes”* (Andrew, 68, male). |
| Barriers for participation | Environmental barriers were transportation and the weather. Participants found it difficult to get to the location by public transport, or to find the nearest parking area if the meeting point was on campus:  *“ (.) it is time-consuming. And it is actually difficult to get here. I have to take a bus. A car park is about 20 minutes away, so it is physically difficult. On one Saturday I couldn’t get in [to the campus] and I spent about half an hour waiting for someone to get me out and let me in”* (Andrew, 68, male).  Personal barriers included lack of time associated with family and community celebrations, holiday, home refurbishments, and carer responsibilities:  *“Yes, it is difficult. You have to decide which is the priority, don’t you? Are you the priority in walking in the group, or what is your consequence of doing the priority? You just have to decide what the priority is”* (Alison, 75, female). |
| ***Theme 2. Walking sessions*** |  |
| Content and delivery | Participants enjoyed the variable routes and walking routes, many of which were undiscovered for some: *“I have discovered loads of different things in [referring to the walk in the park] I have not been to (.)“*(Kate, 62, female).  Stretching exercises were viewed very positively. They encouraged participants to explore different exercises and improved the feeling of support and self-esteem due to a feeling of mastery: *“Yes, initially I thought that when we stopped to do some [Kate: exercises], oh my God, I can’t do that, but I was (.) obviously there was more of us to do this and I saw you doing it and I thought if they can do it (everyone laughs) then I can do it, yeah. Yes, so that was (.) you can really get into the way of thinking you can’t do things, whereas actually you can do that, or you can try it, yeah”* (Sarah, 76, female). |
| Health effects or benefits | Overall, participants had very positive views of the walking intervention, naming among the benefits of walking, its cost-effectiveness in terms of the economic benefits for older adults who lived their adult lives as single parents: *“And because I am on a fixed income now, you know I can’t just go out and earn a bit more money to do something, it does limit you a little bit in what you can do and you have got all this time, but you haven’t got the money. Err, you know, and I brought up a child on my own so she took quite a lot of my salary when I was working [Kate laughs], you know I have never got a lot of money (all laugh) to do what I would really like to do. So, you have to work within that (.)”* (Alison, 75, female). |
| Suggestions to improve | It was suggested to organise walks for different activity levels (e.g. beginners, improvers, advanced level): *“So, you know when the Researcher does the assessment and the Researcher asks you to walk backwards and forwards, how fast can you walk and you do that. And at that point you could divide into three (categories), what people are comfortable at doing “*(Alison, 75, female). |
| ***Theme 3. Healthy workshops*** |  |
| ***Delivery*** | The healthy workshops as delivered were very useful for discussing the topics of interest as well as for bonding and making friendship connections:  *“The more we meet each other, the more we discuss things, we can see that we have the same likes, the same dislikes, so it will become (.) the friendship hopefully will be established and maybe yeah, it will be great support. We will be supporting each other”* (Kate, 62, female). |
| ***Content*** | The loneliness topic has drawn a particular interest of participants among others: *“I think it was a sort of light bulb moment you know, realising that actually we spend a lot of time alone [Kate: but not lonely], and there can be a resentment about that because I have to do everything. If the lawn needs cutting I have to cut it, if someone is coming for lunch (.) I don’t have somebody to help me to prepare lunch (.) it (.) you know, everything is done on my own! I have to do everything (.)”* (Alison, 75, female). |
|  |  |
| ***Suggestions to improve*** | It was suggested to devote a separate day for attending the healthy workshops (rather than immediately after walking sessions) and invite external experts to discuss these topics and share opinions: |
|  | *“Maybe, I would suggest if that’s the case we have a separate time for discussion rather than walking, I agree with you (referring to Alison) because I last time after we have been at [the place for a walk], we were talking about food rather than the discussion topic (.)”* (Kate, 62, female). |
|  | Participants suggested it would be beneficial to use a less structured approach to workshops, make them shorter in duration and more precise:  *“I think it should be more casual, laid back and more general you know, the walk loosens everybody up, puts them in the good mood hopefully, and then ermm you just come back, have a cup of coffee and maybe like Andrew said, half an hour, an hour talks”* (Ben, 65, male). |

Notes: Participants were given pseudonyms.
