# Supplementary material for: Physical Activity Intervention for Loneliness (PAIL) in community-dwelling older adults: a randomised feasibility study
Source: Pilot Feasibility Stud. 2020 May 23;6:73. doi: 10.1186/s40814-020-00587-0 (PMC7245022; doi:10.1186/s40814-020-00587-0)
Supplement: Supplementary file 5 — Additional file 5. Main themes and emerged sub-themes from the end-point focus group interviews [file 40814_2020_587_MOESM5_ESM.docx]

**Additional file 5** Main themes and emerged sub-themes from the end-point focus group interviews

| **Themes and sub-themes** | **Quotes** |
| --- | --- |
| ***Theme 1. Study design and recruitment*** |  |
| *Benefits of participations* | Motivation to do things was one of the main reasons to join the study which was improved after participation in the intervention:  *“You have to just try and keep motivating [yourself], just keep going I suppose, rather than just sitting at home. I don’t know how (.) for me like, I am working four days a week at the moment, so I don’t know how I would feel when I retire, which is going to happen next year so (.)”* (Ben, male, 65). |
|  | Participants felt that participation in the intervention helped them to become more physically active healthy, which was their aim:  *“Ermm, well it has driven home you have to make things happen, you can’t just sit at home. I suppose the only thing is just the physical you know, what comes with age is just the physical side and not be able to do quite as much, so that I suppose is something about so (.)”* (Ben, male, 65). |
| *Barriers* | Environmental barriers were season of the year, transport difficulties and walking routes. In the opinion of most participants, it would be best to start the walking intervention is in the spring throughout the summer season:  “*I think it started really too early in the year, because (.) [Andrew: Yeah, and it was winter] and it was winter. So, it would have been better really to start it maybe in spring. More people will be interested in joining* (.)” (Ben, male, 65). |
|  | Among personal barriers were time of the intervention coincident with use of public transport, late night hobbies and health reasons. It was suggested that an 11:00 start was best for most participants:  *“(.) in our age in the morning we are not fast. It takes time in the morning. We cannot rush. Then on top of it we could take medication you know, like everybody (.) I take triple tablets and you can’t just (.) it takes time”* (Nick, male, 73). |
| *Improvements made* | Improvement since the beginning of the PAIL intervention included an increase in participant number, as well as participants being more diverse:  *“We had yes, we had met friends. And the most (.) the thing is mixing with different age groups and different people, yeah. This definitely helps. I meet with different people throughout the season”* (Nick, male, 73). |
|  | The optimal number of people in the group, in the opinion of the participants, is 6 to 12 people to allow participants to repeatedly socialise with each other and establish friendship connections:  *“This is probably about right. What is it, seven or eight?”* (Ben, 65, male).  *“Yes, seven or* *eight”* (Andrew, 68, male)*.* |
|  | With the number of people increased, social interaction improved as well:  *“More interaction, it has been more social…I mean when we went for a coffee or things like that. I mean yesterday, last two times it has been like, we have gone for coffee it has been quite pleasant, isn’t it?”* (Ben, 65, male). |
| ***Theme 2. Walking sessions*** |  |
| *Benefits* | The benefits of walking sessions from the end-point focus group interviews included social engagement:  *“Enjoyed the walks, enjoyed the conversations with people that we have met on the walks (.) Leo, Nick [referring to participants by names] so yeah, it’s been interesting. And it, sort of the conversations evolve (the topic that the Researcher gives us) it evolves into things (laughs) that (.) which is fun, yeah (.)”* (Sarah, female, 76). |
|  | Participants especially valued stretching exercises for health reasons:  *“I enjoyed the exercise thing. It is quite, you know (.) it is just (.) She sort of said, you know, made us aware of sort, of what sort of exercise is good for, what parts of your body and so forth. So, I mean it is important, isn’t it to keep moving, keep active and this is what part of the programme is about, isn’t it?”* (Ben, male, 65). |
| *Improvements made* | Walking routes became more diversified compared to the beginning of the PAIL intervention:  *“I mean there is a whole programme now. She [the Researcher] organised it all now”* (Ben, 65, male)*.*  *“Yeah, she works out where we are walking for the next three months almost”* (Andrew, 68, male)*.* |
|  | Intervention participants were offered the opportunity to continue walks based on their concern about the follow-up as followed from the mid-point interviews:  *“It will be interesting to see whether at the end there will be any continuation (.) I mean already you can see like one lady, the lady seems to get very friendly very quickly, and there is one lady already bringing one in and picking her up from [location] or the rest of it, so yeah, it seems to be (.)”* (Ben, male, 65). |
| *Suggestions for improvement in the future intervention* | Organise groups by different abilities:  *“I think possibly from people that are joining for an exercise point of view, people’s abilities need to be looked at (.). What they are capable of physically. So, if it’s, if you got people that walk regularly and walk quite quickly, you may also have people who walk very slowly (.)it can be a little bit, not frustrating but you know, the difference is too much”* (Sarah, female, 76). |
| ***Theme 3. Healthy workshops*** |  |
| *Benefits* | Benefits of participation in the healthy workshops were social engagement and opportunity to meet others, as well as sharing opinions regarding the topics of interest:  *“(.) we had a chance to talk about something interesting (for me it was very interesting) about our dental hygiene and it is an important thing to remember to take care of (.) to get into any community and to say start the conversation or to be involved into the conversation (.) I think all of us, we know what I am talking about to avoid bad smell (referring to the breath smell) so hygiene is really important, a vital thing I would say, to mention our last subject of our discussion”* (Leo, male, 60). |
|  | First friendship gains started from as early as the second or third walk:  *“On the first session we had introduced each other anyway, so we started to know each other maybe at the second (session)”* (Nick, male, 73). |
| *Improvements made during the project* | Among improvements made was a weekly social programme and information about local social events:  *“Well I suppose that to try organise some social programmes, I suppose. But to be honest, [the Researcher] has widened it out, hasn’t she? She seems to have been organising quite few things. She mentions along the way, “you know you could do this, or this is available” (,) so she is putting in (.) there is a little bit of input”* (Ben, male, 65).  Changed the location of healthy workshops:  *“I think the things did notably change after the mid-focus group because after that, then it was less classroom based [activities] and then basically we were out more. That dropped completely almost, wasn’t it? [Andrew: Yeah] But now it is the beginning of the next group, so the Researcher is repeating the procedure except it is not classroom-based now, it is outside-based”* (Ben, male, 65). |
|  | The delivery approach was changed after the mid-point focus groups from note or slides-led to a less formal approach where participants were more engaged in a conversation about the topic and beyond over hot beverages:  *“I could feel relaxed and just enjoyed walks and discussion [Sarah: Yeah, yeah]”* (Leo, male, 60)*.* |

Notes: Participants were given pseudonyms.
