# Supplementary material for: Physical Activity Intervention for Loneliness (PAIL) in community-dwelling older adults: a randomised feasibility study
Source: Pilot Feasibility Stud. 2020 May 23;6:73. doi: 10.1186/s40814-020-00587-0 (PMC7245022; doi:10.1186/s40814-020-00587-0)
Supplement: Supplementary file 6 — Additional file 6. Weekly average attendance rate (%) for 12 weeks walking intervention [file 40814_2020_587_MOESM6_ESM.docx]

**Additional file 6** Weekly average attendance rate (%) for 12 weeks walking intervention
