# Supplementary material for: Physical Activity Intervention for Loneliness (PAIL) in community-dwelling older adults: a randomised feasibility study
Source: Pilot Feasibility Stud. 2020 May 23;6:73. doi: 10.1186/s40814-020-00587-0 (PMC7245022; doi:10.1186/s40814-020-00587-0)
Supplement: Supplementary file 7 — Additional file 7. Sample size calculations for psychosocial outcomes [file 40814_2020_587_MOESM7_ESM.docx]

**Additional file 7** Sample size calculations for psychosocial outcomes

| **Variables** | **Observed effect size** η^2^ **for group x time interaction** | **Observed p value** | **Correlation among repeated measures** | **Achieved power (1-β) (N=25, 2 groups,**  **2 time points)** | **N required for significant effects in future with 1-β = .80, α = .05** | **N required accounting for 20% attrition** |
| --- | --- | --- | --- | --- | --- | --- |
| Loneliness | .014 | .57 | 0.75 | 0.88 | 72 | 87 |
| Social support | .030 | .41 | 0.65 | 0.90 | 48 | 58 |
| LSN (Total) | .026 | .44 | 0.70 | 0.91 | 48 | 26 |
| LSN (Family) | .030 | .71 | 0.78 | 0.99 | 30 | 36 |
| LSN (Friends) | .011 | .26 | 0.83 | 0.75 | 62 | 74 |
| HADS (Depression) | .008 | .67 | 0.23 | 0.74 | 378 | 454 |
| HADS (Anxiety) | .032 | .39 | 0.46 | 0.81 | 68 | 82 |
| SEE | .122 | .09 | 0.66 | 1.00 | 12 | 14 |
| SSC | .000 | .97 | 0.83 | - | - | - |
| Expected outcomes | .033 | .38 | 0.51 | 0.84 | 60 | 72 |
| Barriers for exercise | .011 | .62 | 0.52 | 0.78 | 172 | 206 |

Abbreviations: N- total sample size, LSN – Lubben’s social networks, HADS – Hospital Anxiety and Depression, SEE – self-efficacy for exercise, SSC – satisfaction with social contacts.
